# Enhancing photothermal therapy of tumors with image-guided thermal control of gene-expressing bacteria

**DOI:** 10.7150/thno.98257

**Published:** 2024-09-09

**Authors:** Fengyi Zeng, Meng Du, Yaozhang Yang, Jinghui Fang, Yuanyuan Wang, MeeiChyn Goh, Yan Lin, Huaiyu Wang, Fei Yan, Zhiyi Chen

**Affiliations:** 1 Key Laboratory of Medical Imaging Precision Theranostics and Radiation Protection, College of Hunan Province, the Affiliated Changsha Central Hospital, University of South China, 161 Shaoshan South Road, Changsha, Hunan Province (China).; 2 Institute of Medical Imaging, Hengyang Medical School, University of South China, 28 Changsheng West Road, Hengyang, Hunan Province (China).; 3 Department of Medical Imaging, The Affiliated Changsha Central Hospital, Hengyang Medical School, University of South China, 161 Shaoshan South Road, Changsha, Hunan Province (China).; 4 CAS Key Laboratory of Quantitative Engineering Biology, Shenzhen Institute of Synthetic Biology, Shenzhen Institute of Advanced Technology, Chinese Academy of Sciences, 1068 Xueyuan Avenue, Shenzhen, Guangdong Province (China).; 5 Department of Ultrasound Medicine, Third Affiliated Hospital of Guangzhou Medical University, 63 Duobao Road, Guangzhou, Guangdong Province (China).; 6 Center for Human Tissues and Organs Degeneration, Shenzhen Institutes of Advanced Technology, Chinese Academy of Sciences, 1068 Xueyuan Avenue, Shenzhen, Guangdong Province (China).

**Keywords:** Bacteria mediated tumor therapy, contrast-enhanced ultrasound imaging, multimodal imaging, gas vesicles, photothermal therapy

## Abstract

**Purpose:** Bacteria-mediated tumor therapy has showed promising potential for cancer therapy. However, the efficacy of bacterial monotherapy treatment which can express and release therapeutic proteins in tumors has been found to be unsatisfactory. To date, synergistic therapy has emerged as a promising approach to achieve stronger therapeutic outcomes compared to bacterial monotherapy. It is a challenge to visualize these tumor-homing bacteria *in vivo* and guide them to express and release in situ therapeutic proteins.

**Procedure:** We have developed a kind of engineered bacteria (named CGB@ICG) genetically incorporating acoustic reporter proteins and thermo-inducible ClyA expression gene circuit and chemically modified with indocyanine green on the bacterial surface. The presence of acoustic reporter proteins and indocyanine green facilitates the visualization of CGB@ICG via contrast-enhanced ultrasound imaging and optical imaging, making it possible to guide the sound wave or laser to irradiate precisely these bacteria for inducing the expression of ClyA protein via acoustic- or photothermal effects. The expression and secretion of ClyA protein in the tumor, combined with photothermal therapy, greatly enhanced the anti-tumor efficacy of the engineered bacteria and improved their biosafety.

**Results:** We successfully performed multimodal imaging of CGB@ICG *in vivo* resulting in remoting control the expression of ClyA protein in tumor. *In vivo* experiments showed that bacteria-mediated therapy combined photothermal therapy exhibited a rapid decrease in tumor volume compared to other groups, while the tumor volume of the combination therapy group continued to decrease and even achieved complete healing. Thus, combination therapy not only reduced the rate of tumor growth but also prevented the proliferation of tumor cells for an extended period.

**Conclusion:** Our study demonstrated that CGB@ICG serves as an efficacious imaging agent and delivery vector to combine engineered bacteria with photothermal therapy, holding great promise for tumor treatment.

## Introduction

The utilization of bacteria as delivery vectors has been regarded as a promising strategy for drug delivery against cancer due to several inherent advantages 1-3. Most researchers have found that non-pathogenic *E. coli* possess the capability to preferentially target and colonize the solid tumor core individually 4, 5. This phenomenon arises from the nutrient-rich and lower oxygen levels within the solid tumor core, which provides a conducive environment for bacterial proliferation 6-8. Furthermore, bacteria offer the feasibility and controllability to express therapeutic agents in situ through genetic editing and modification 9, 10. The abundance of functional groups on bacterial surfaces allows for functionalization, enabling the development of novel drug delivery strategies 11. Moreover, bacteria, acting as natural antigens, exert immunostimulatory effects, directly activating immune responses.

Advancements in synthetic biology have empowered researchers to ingeniously design engineered bacteria carrying plasmids expressing therapeutic agents, significantly enhancing the effectiveness of anti-tumor treatments 12-14. For instance, Cytolysin A (ClyA), a 34-kDa protein capable of forming pores, is naturally found in bacteria and can arrest the cell cycle at G2/M phase 15. The study has surmised that ClyA protein created perforations in the cell membrane, resulting in the cell swelling and the subsequent release of intracellular contents into the surrounding microenvironment, ultimately leading to cell death 16. However, when administrated intravenously, ClyA protein tends to primarily localize in the liver and spleen. To mitigate systemic toxicity, it is crucial to regulate the expression of ClyA protein specifically in target lesions. Numerous studies have developed various inducible gene expression systems in bacteria to ensure a substantial concentration of ClyA protein in target lesions. These systems are designed to sense various signals to trigger promotors for gene expression, including chemical and biological inducers, and physical stimulation 9, 17. For instance, several researchers have constructed inducible promoters activated by arabinose 18 or doxycycline to control ClyA protein expression 19. However, gene expression induced by chemical inducers is regulated by the concentrations of the inducers and the timing of their administration. Due to the low specificity of chemical inducers for tumor targets, they may remain at relatively low levels in tumor sites, which may not effectively induce bacterial gene expression 20. An alternative method involves the use of biological inducers, such as synchronized lysis circuits, where bacteria can release therapeutic products as they reach critical density 21-23. Nevertheless, confirming the density of bacteria in the local tumor in live animals remains challenging. Thus, physical stimulation methods such as radiation and heating are utilized to remotely trigger gene expression. However, radiation may cause unavoidable DNA damage to normal tissues surrounding the tumor 24, 25. To date, an alternative inducible plasmid, pBV220, utilizes the thermal-inducible pR and pL promoters, which are triggered to express the protein at temperatures between 42-45 °C 26. However, conventional heating methods can easily damage the surrounding normal tissues due to the inevitably large heating area. Therefore, finding a more precise heating method is necessary. Recently, brief hyperthermia by high intensity focused ultrasound (HIFU) has been reported to promote gene expression in the thermal-inducible system 27. This may because HIFU offers a focused ultrasound wave with high energy, which can damage the target area without affecting surrounding tissues 28. Notably, with the development of nanotechnology, numerous nanomaterials have been applied to work with engineered bacteria to promote the in-situ production of therapeutic products 29, 30. A thermally sensitive inducible system comprising gold nanoparticles and bacteria has been reported to precisely control the expression of TNF-α or ClyA protein. These systems are triggered by the heat transferred from gold nanoparticles upon near-infrared (NIR) laser irradiation to express therapeutic protein 16, 31. Furthermore, an inducible system with a radiation-sensitive promotor has been developed, which combines the therapeutic effects of radiation therapy with inducible therapeutic protein 32. Therefore, ultrasound-controllable or laser-controllable bacterial systems can be applied to significantly express ClyA protein and suppress tumor growth 33.

Despite advances in inducible bacterial systems for tumor therapy, recent studies have underscored the presence of significant individual variances in therapeutic outcomes. This discrepancy primarily arises due to the absence of *in vivo* methodologies for the visualization of bacteria, potentially leading to the oversight of maximal bacterial accumulation within tumor lesions. Consequently, this oversight can impact the expression of therapeutic proteins and the evaluation of therapeutic efficacy. Therefore, the development of various molecular imaging techniques is imperative to facilitate the *in vivo* visualization of engineered bacteria, thereby confirming the inducible timing of gene expression following precise delivery to tumor lesions. Among these techniques, fluorescence imaging (FL), photoacoustic imaging (PAI), and ultrasound imaging (US) are notable. Fluorescence imaging, particularly utilizing long-wavelength NIR (700-1700 nm), can achieve high-resolution imaging in deep tissues by minimizing photon scattering and enhancing light absorption relative to short-wavelength light (360-760 nm) 34. However, the quest for high-performance fluorophores remains a challenge in NIR fluorescence imaging. Conversely, PAI offers superior imaging resolution owing to the reduced tissue scattering of ultrasound as opposed to light, and it outperforms most optical imaging methodologies in terms of depth penetration. While exogenous PAI probes can be conjugated with bacteria for *in vivo* monitoring, the presence of free PAI probes may influence the accurate assessment of bacterial accumulation in tumor lesions. Although optical imaging techniques show great promise in preclinical settings, their clinical application has been limited. In clinical practice, ultrasound imaging especially contrast-enhanced ultrasound imaging (CEUS) can dynamically and efficiently display microcirculation perfusion in real time by contrast agents, which has the advantages of high spatial-temporal resolution and minimal radiation exposure 35. However, existing methods for imaging bacterial location and function, primarily based on optical reporter genes, exhibit limited deep tissue performance. To date, the introduction of acoustic reporter genes has enabled* in vivo* monitoring of bacteria via ultrasound 36. Reports indicate that the expression of acoustic gene clusters (ARGs) resulted in *E. coli* containing gas vesicles (GVs), providing imaging performance beyond expectation 37. Nonetheless, the sensitivity of ultrasound imaging still requires enhancement. All imaging modalities present both advantages and disadvantages 38. Considering the complementary performance of different imaging technologies, multimodal imaging may represent an effective approach for accurately visualizing the delivery of bacteria *in vivo*.

Despite various studies have been conducted in tumor therapy with outstanding results and most studies focused on one aspect of bacteria-mediated tumor therapy (BMTT) 39. Therefore, we have consolidated tumor targeting, payload delivery, and controlled gene-releasing into one biohybrid bacterial system to monitor delivery and achieve efficient tumor suppression. In this study, we engineered an image-guided, thermally controlled bacterium (CGB@ICG), facilitating precise imaging and treatment modalities. We firstly transformed two plasmids into engineered bacteria to construct engineered bacteria with a two-plasmid system (CAB). Next, the expression of acoustic reporter gene clusters, induced by chemical inducers of IPTG and L-Ara, resulted in the engineering of bacteria containing GVs suitable for ultrasound imaging (CGB). Subsequently, photothermal indocyanine green (ICG) was conjugated to the surface of CGB using amide bond to construct CGB@ICG. The *in vivo* monitoring of CGB@ICG was enhanced through the application of fluorescence, photoacoustic, and/or ultrasound imaging techniques, confirming the inducible timing of ClyA protein expression within tumor lesions. Upon the accumulation of CGB@ICG in the tumor, either an NIR laser or HIFU was employed to irradiate and heat the bacteria, thereby triggering the secretion of ClyA protein, which can arrest the cancer cell cycle. To further inhibit tumor metastasis and eradicate intratumoral bacteria, a second NIR laser irradiation was administered. The resultant biohybrid bacterial system demonstrated the outstanding multimodal imaging capability and bacteria-mediated photothermal therapeutic efficacy.

**Figure 1:**
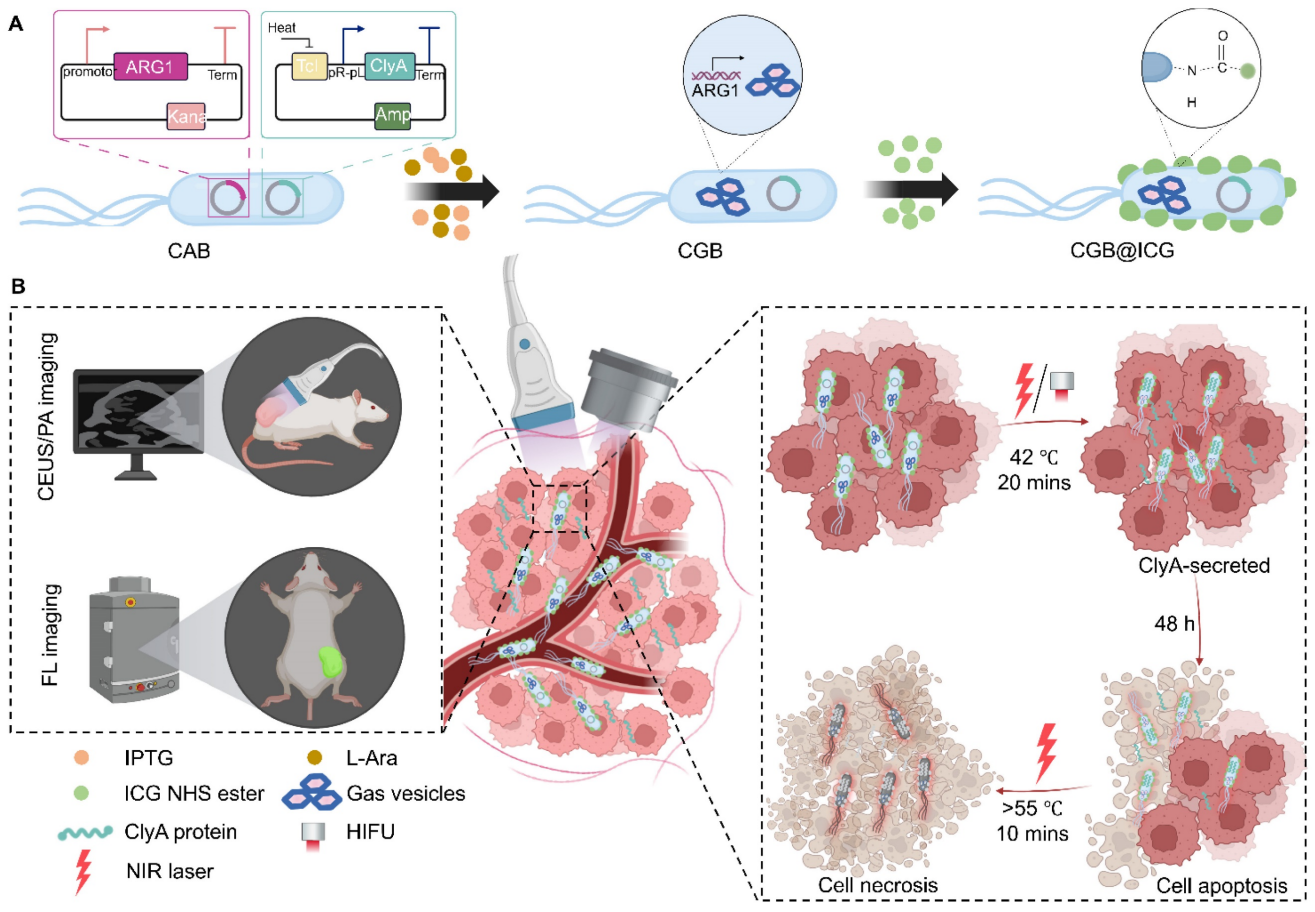
Illustration of image-guided thermal-controlled therapeutic bacteria nano-system designed to enhance photothermal therapy in tumors. (A) Synthesis of the bacteria nanosystem (CGB@ICG) through the conjugation of indocyanine green (ICG) with ClyA-GVs expressing bacteria. The expression of GVs was induced by isopropyl β-D-1-thiogalactopyranoside (IPTG) and L-Ara, while ICG was conjugated to the bacterial surface via an amide bond. (B) *In vivo* tracking of CGB@ICG migration into tumor cores is facilitated by US/PA/FL imaging, enabling precise thermal regulation of ClyA secretion within the tumor, thereby promoting cell apoptosis. Subsequent NIR laser irradiation enhances necrosis within the tumor.

The construction process of CGB@CIG and its therapeutic principle are illustrated in Fig. 1. The pET28a_T7-ARG1 plasmid containing an arabinose promoter and T7 promoter was transferred into bacteria, and the pBV220-ClyA plasmid containing pR-pL promoter was also transferred into bacteria to construct chassis bacteria (CAB). Next, the pET28a_T7-ARG1 plasmid could be induced by IPTG and L-Ara to produce GVs, which are gas-filled protein-shelled contrast agents to construct GVs overexpressed bacteria (CGB). The GVs has been proven as ultrasound contrast agents for contrast-enhanced ultrasound imaging. Following, photothermal imaging agent (ICG) was bond to the surface of CGB (CGB@ICG), which showed good imaging capability and thermally sensitivity. After monitoring the accumulation and penetration of CGB@ICG in tumor, CGB@ICG could be triggered under thermal effects to express ClyA protein because the structure of pR-pL promoter in pBV220-ClyA plasmid can be change at temperatures between 42-45 °C. Meanwhile, combination therapy greatly enhanced the antitumor efficacy of the engineered bacteria and improved their biosafety. Collectively, our findings demonstrate that image-guided, thermally controlled bacteria offer a novel approach whereby the combination of photothermal therapy (PTT) and thermal-induction ClyA protein expression substantially inhibits tumor progression.

## Materials and Methods

### Materials

The pBV220-ClyA and pET28a_T7-ARG1 (Addgene plasmid #106473) plasmids were acquired from the CAS Key Laboratory of Quantitative Engineering Biology. The *E. coli* BL21(AI) strain was purchased from Angyubio Co., Ltd. (Shanghai), while Indocyanine Green was supplied by Ruixibiotech Co., Ltd. (Xian). LB Agar and LB broth media were purchased from Huankai Co., Ltd. (Guangdong), whereas isopropyl β-D-1-thiogalactopyranoside (IPTG) and L-arabinose were obtained from Macklin Co., Ltd. (Shanghai). Glucose was sourced from Sangon Co., Ltd. (Shanghai). Kanamycin and ampicillin were procured from Solarbio Co., Ltd. (Beijing). The 4T1 cells, a mouse breast cancer cell line, were obtained from the American Type Culture Collection (ATCC). DMEM culture medium and PBS were acquired from Procell Co., Ltd. (Wuhan). The penicillin-streptomycin, fetal bovine serum (FBS), and trypsin were obtained from Gibco (USA). The Cell Counting Kit-8 was sourced from Dojindo (Typo). The Calcein-AM/PI double staining kit was purchased from Yeasen Co., Ltd. (Shanghai).

### Preparation of CGB transformed bacterial strains

The pBV220-ClyA plasmid was transformed into *E. coli* BL21(AI) competent cells using a chemical transformation protocol. Subsequently, the cells were cultured in LB agar broth medium supplemented with ampicillin at 37 °C for 24 hours to isolate positive ClyA-BL21(AI) (CB) clones. Then, CB cells were then rendered competent and transformed with the pET28a_T7-ARG1 plasmid using an electroporation protocol. These cells were cultured in LB agar broth containing both ampicillin and kanamycin at 37 °C for 24 hours to select for positive ClyA-ARG1-BL21(AI) (CAB) clones. Following selection, CAB cells were inoculated into 5 mL of LB broth medium supplemented with 50 µg/mL kanamycin and 200 µg/mL ampicillin and incubated in a shaking incubator at 37 °C until the optical density at 600 nm (OD_600nm_) reached 0.5. The culture was then transferred into 5 mL of LB broth containing 50 µg/mL kanamycin, 200 µg/mL ampicillin, and 1% glucose and incubated under the same conditions for 16 hours. Subsequently, the cells were diluted at a 1:100 ratio into 1 L of LB broth medium containing 50 µg/mL kanamycin, 200 µg/mL ampicillin, and 0.2% glucose, and incubated at 37 °C until the OD_600nm_ reached 0.5. At this stage, 0.5% L-arabinose and 0.4 mM IPTG inducers were added to the medium, and the cells were further incubated at 30 °C for 24 hours. Finally, the cells were centrifuged at 350 × *g* for 4 hours to collect the GV-producing cells, from which ClyA-GVs-BL21(AI) (CGB) was derived for further experimental analysis.

### Covalent coupling of ICG to CGB using amide bond

CGB cells were cultured in LB broth medium at 37 °C until reaching an OD_600nm_ of 0.5. Following this, the medium was supplemented with ICG, and the bacterial suspension was incubated at room temperature for 12 hours. During this period, the yellow-colored solution gradually turned green, indicating the coupling of ICG to the CGB cells. Following incubation, the cells were washed three times with PBS, using centrifugation at 4,500 rpm for 4 minutes for each wash, and then resuspended in PBS to obtain CGB@ICG. This method was consistently applied to prepare CAB@ICG or GB@ICG cells, ensuring uniformity across cell types.

### Characterization of CGB@ICG

Transmission electron microscopy (JEM-1400), confocal microscopy (Eclipse Ti), and phase contrast microscopy (IX83) were used to observe the morphological structures of CAB@ICG and CGB@ICG bacteria. Dynamic light scattering (Malvern Zetasizer Nano ZS) was utilized for measuring the zeta potential of ICG, CGB, and CGB@ICG. The absorption spectra of ICG, CGB, and CGB@ICG were measured using a multimode microplate reader (M1000PRO), and the absorption spectra and coupling efficiency of CGB@ICG were evaluated through absorbance measurements.

### Photothermal properties of CGB@ICG

The investigation into photothermal properties of GCB@ICG involved measuring temperature change among CGB, ICG, and CGB@ICG under NIR laser irradiation. An 808 nm NIR laser, with a power density of 1 W/cm^2^, was utilized to irradiate various concentrations of CGB@ICG (10^8^, 5×10^8^, 10^9^ CFU/mL) for a duration of 5 minutes, employing PBS as the control group. The temperature variation of CGB@ICG (5×10^8^ CFU/ml) was recorded across different concentrations of ICG (0, 10, 25, 75 μg/mL) and varying power density (0.5, 0.75, 1, 1.5 W/cm^2^). Additionally, CGB@ICG (5×10^8^ CFU/mL; 50 μg/mL ICG) underwent irradiation using an 808 nm NIR laser at a power density of 0.75 W/cm^2^ to evaluate its repeated photothermal performance. Similar process was performed to observe the temperature change of CGB@ICG irradiated by HIFU irradiation. The temperature changes of the samples were continuously monitored at 30-second intervals using an FLIR E4 infrared camera.

### Thermally-induced ClyA expression of CGB@ICG

CGB@ICG solutions were prepared and cultured in LB broth medium, supplemented with 200 μg/mL ampicillin and 50 μg/mL kanamycin, and incubated at 30 °C until the colonies reached a concentration of 5×10^8^ CFU/mL. For the analysis of temperature-dependent expression, three shaker-incubators were loaded with 5 mL of the CGB@ICG solution, each having an optical density (OD_600nm_) of 0.5, and were set to 30 °C, 37 °C, or 42 °C for a duration of 40 minutes. In the time-dependent expression studies, a shaker-incubator containing 5 mL of the CGB@ICG solution, at a concentration of 5×10^8^ CFU/mL, was maintained to 42 °C for various durations (0, 10, 20, 30 and 40 minutes) via thermal effects. Subsequently, all samples were centrifuged to facilitate precipitation and then mixed with a loading buffer (P0015, Beyotime), followed by incubation at 100 °C for 15 minutes. The samples were further centrifuged at 13,300 rpm for 20 minutes. Finally, the precipitates were analyzed using sodium dodecyl sulfate-polyacrylamide gel electrophoresis (SDS-PAGE). To evaluate the expression of ClyA protein via HIFU irradiation, the incubators were irradiated by HIFU and remained at 42-45 °C.

### *In vitro* imaging of CGB@ICG

Imaging phantoms were prepared by melting 1% (w/v) agar in distilled water and casting wells using 1.5 ml EP tube. CGB@ICG (5×10^8^ CFU/mL) was imaged under induction by various concentrations of IPTG, ranging from 0.2 mM to 1 mM. Prior to sample loading, the concentration of CGB@ICG was determined by measuring bacterial concentrations, which ranged from 5×10^7^-10^9^ CFU/mL, with CAB@ICG serving as the control group. The ultrasound imaging performance of CGB@ICG and CAB@ICG was evaluated using VisualSonics Vevo2100 (VisualSonics, Inc., Toronto, Canada). Contrast-enhanced ultrasound image acquisition was conducted with a center frequency of 21 MHz and power of 20%. Additionally, the photoacoustic and fluorescence imaging capabilities of CGB@ICG were assessed using VisualSonics Vevo LAZR-X (VisualSonics, Inc., Toronto, Canada) and IVIS Spectrum (PerkinElmer, Waltham, MA, USA), respectively. Signal and photoacoustic intensities were quantitatively analyzed using ImageJ software, while the total flux was analyzed using the IVIS Spectrum's built-in signal processing software.

### Establishment of a tumor-bearing mouse model

Eight-week-old female BALB/c mice were acquired from Bestest Biotechnology Co. (Zhuhai, China). The animal experiments received approval from the Institutional Animal Care and Use Committee (IACUC) of the Animal Experiment Center of Shenzhen Institutes of Advanced Technology, Chinese Academy of Sciences (SIAT-IACUC-190620-YGS-YF-A0766). All procedures complied with the ARRIVE guidelines and were conducted in accordance with the National Research Council's Guide for the Care and Use of Laboratory Animals. Breast cancer (4T1) cells were cultured with DMEM supplemented with 10% FBS and 1% penicillin-streptomycin and maintained at 37 °C in a 5% CO_2_ humidified atmosphere. The cultured 4T1 cells were trypsinized, suspended in PBS solution, and subcutaneously injected into the right flank of the mice (1×10^6^ cells in 0.1 mL per mouse) to establish the tumor-bearing model. Tumors generally reached an appropriate size after seven days. Tumor volume was calculated using the formula of length × width^2^. Mice were euthanized when tumors reached 2,000 cm^3^.

### *In vivo* multimodal image-guided visualization of CGB@ICG

Two groups, designated as CAB@ICG and CGB@ICG, were subjected to intratumor injection imaging experiments. Each group received an injection of 110 μL of CAB@ICG or CGB@ICG, at a concentration of 5×10^8^ CFU/mL. Tumor images were captured both before and after the injection using various multimodal imaging techniques. For the intravenous injection imaging experiments, initial tumor images were obtained using multimodal imaging modalities prior to administering the bacteria samples through the tail vein. This was followed by the injection of 110 μL of either CAB@ICG or CGB@ICG (5×10^8^ CFU/mL), after which images were again acquired. Twenty-four hours subsequent to the initial injection, tumor images were captured in the same plane as the previous images, with this procedure being repeated for two additional bacterial injections. Throughout all* in vivo* imaging experiments, ultrasound imaging and PAI were conducted at a frequency of 21 MHz and a power setting of 20%, respectively. Fluorescence imaging was performed at excitation/emission wavelengths of 745/820 nm. The signal and photoacoustic intensity were quantitatively analyzed using ImageJ software, while the total flux measurements were conducted using the IVIS Spectrum's built-in signal processing software.

### *In vitro* cell cytotoxicity experiments

A total of 2×10^4^ 4T1 cells were seeded in each well of a 96-well plate and cultured in 100 μL DMEM medium supplemented with 10% FBS for 12 hours. The experimental groups—GB@ICG+HIFU, CGB@ICG+HIFU, CGB@ICG+HIFU+L, GB@ICG+L, CGB@ICG+L, and CGB@ICG+L+L—were subject to irradiation using an 808 nm NIR laser or HIFU for 20 minutes prior to the addition of bacteria to the wells. The temperature was maintained within the range of 42-45 °C. Subsequently, 10 μL of the samples were dispensed into each well and incubated for four hours. After this incubation period, the CGB@ICG+HIFU+L and CGB@ICG+L+L groups received a second round of 808 nm NIR laser irradiation for 10 minutes, with the temperature adjusted to between 55-56 °C. The cells were then incubated for an additional 30 minutes, resulting in a total incubation time of 4.5 hours. The plate was subsequently washed three times with PBS containing 300 μg/mL gentamicin, with each wash lasting 5 minutes 40. To conclude, 100 μL of DMEM medium containing 10% CCK-8 was added to each well, and the cells were incubated for 1.5 hours. The absorbance was measured at 450 nm using a multimodal microplate reader.

The co-incubation procedure mirrored the aforementioned protocol. The 10× Assay Buffer was diluted with deionized water to achieve a 1×Assay Buffer concentration. Then, 5 μL of Calcein-AM solution (2 mM) and 15 μL of PI solution (1.5 mM) were added to 5 mL of 1×Assay Buffer and mixed well to prepare a staining working solution. Finally, 100 μL of this solution was applied to each well and the plate was incubated for 15 minutes. The fluorescence signal from the wells was subsequently examined using a fluorescence microscope.

### Tumor targeting of CGB@ICG

Eight mice were allocated into two groups: live bacteria and dead bacteria, with four mice in each group (n = 4). For the dead bacteria group, CGB@ICG was exposed to 90 °C for 45 minutes. Each mouse, possessing a tumor volume of approximately 100 mm^3^ at the time of bacterial injection, received an intravenous injection of 5×10^8^ CFU/mL of bacteria through the tail vein. The accumulation of bacteria within the tumor was monitored at various time points (0, 12, 24, 48, 72, and 96 hours) utilizing the IVIS Spectrum system. Forty-eight hours post-injection, the mice were euthanized, and tumors and organs (heart, liver, spleen, lungs, and kidneys) were extracted for *ex vivo* imaging using fluorescence imaging techniques. Additionally, tumors and organs of equal weights were placed on ice, subsequently homogenized into a fluid, and 100 μL of this fluid was diluted in a range from 10 to 1,000 times before being spread on agar plates containing 200 µg/mL ampicillin and 50 µg/mL kanamycin. The agar plates were then incubated at 37 °C for 12 hours.

### *In vivo* tumor treatment

4T1 tumor-bearing mice, each with tumors of approximately 100 mm^3^, were randomly assigned to four groups (n = 6): control, CGB@ICG, CGB@ICG+HIFU, and CGB@ICG+HIFU+L. The CGB@ICG, CGB@ICG+HIFU, and CGB@ICG+HIFU+L groups received intravenous injections of 110 μL of CGB@ICG at 5×10^8^ CFU/ml, while the control group received 110 μL of PBS. Forty-eight hours post-injection, the CGB@ICG+HIFU and CGB@ICG+HIFU+L groups underwent HIFU irradiation for 20 minutes, resulting in a tumor temperature of 42 °C. Twenty-four hours post-HIFU irradiation, the CGB@ICG+HIFU+L group underwent an 808 nm NIR laser irradiation for 10 minutes, resulting in a tumor temperature of 55 °C. Tumor volume and body weight of the mice were recorded every other day. On the 26^th^ day, one mouse from each group was euthanized, and tumors and organs were collected for H&E staining, Ki67 staining, and TUNEL assay. The survival curve of the mice was recorded upon euthanasia.

To enhance the antitumor effect of combination therapy, 4T1 tumor-bearing mice were divided into six groups (n = 6): control, GB@ICG, CGB@ICG, GB@ICG+L, CGB@ICG+L, and CGB@ICG+L+L. Mice received intravenous injections of 110 μL of GB@ICG or CGB@ICG at 5×10^8^ CFU/ml. Forty-eight hours post-injection, the GB@ICG+L, CGB@ICG+L, and CGB@ICG+L+L groups underwent 20-minute irradiation with an 808 nm NIR laser. Twenty-four hours post-laser irradiation, the CGB@ICG+L+L group received a second NIR laser irradiation for 5 minutes. Tumor volume and body weight of the mice were recorded every other day. Mice were euthanized on day 26, and tumors and organs were collected and analyzed.

### Biosafety assay

Routine blood biochemical analyses were conducted., and organs were subjected to H&E staining to assess the biosafety profile of CGB@ICG. Tumor-bearing mice were divided into six groups (n=3): control, GB@ICG, CGB@ICG, GB@ICG+L, CGB@ICG+L, and CGB@ICG+L+L. Blood samples were collected via retro-orbital bleeding at days 1 and 14 post-injection. On day 14, the mice were euthanized, and their organs were harvested for H&E staining.

### Statistical analysis

All data are presented as mean ± SD. An independent *t*-test was employed for the two-group comparison, while a two-way analysis of variance using the Tukey post-hoc test was utilized for comparisons involving more than two groups. Statistical analyses were performed using GraphPad Prism 8.

## Results

### Characterization and photothermal properties of CGB@ICG for ClyA protein expression

**Figure 2:**
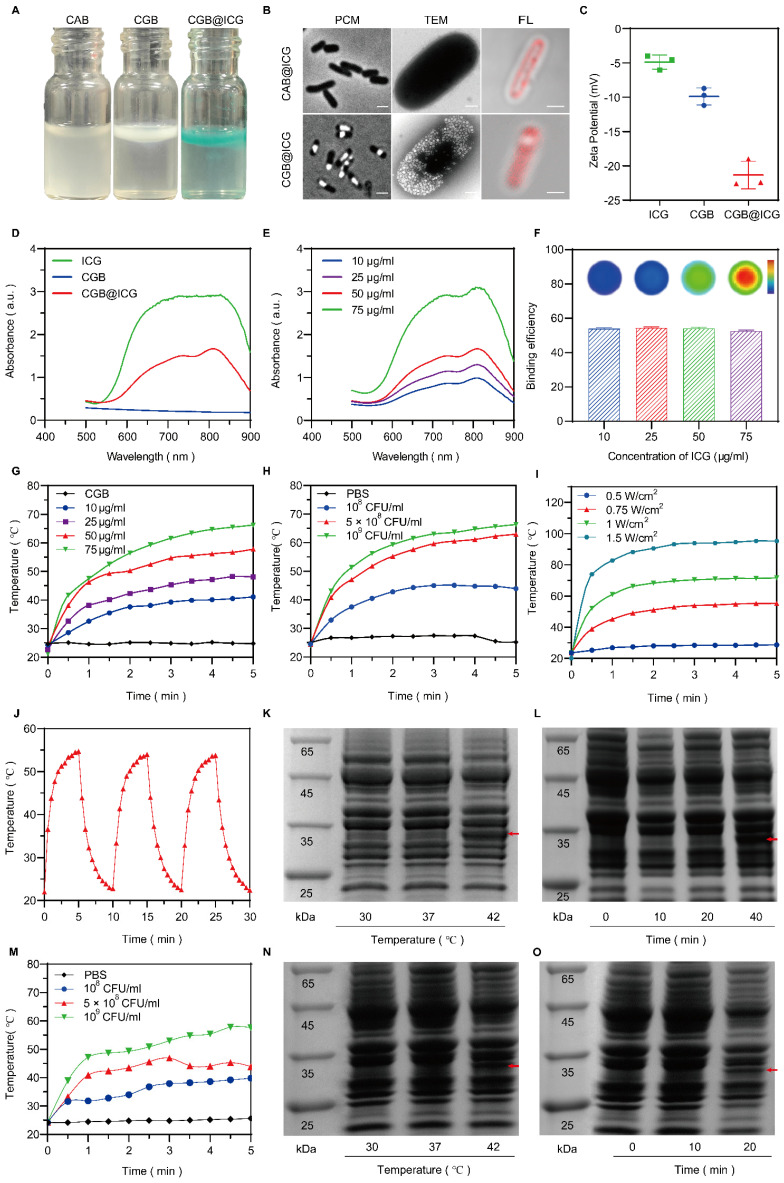
Characterization, photothermal performance, and thermal sensitivity of CGB@ICG. (A) Photographs depicting PBS solutions containing CAB, CGB, and CGB@ICG. (B) Phase contrast images (left, scale bar: 1 μm), TEM images (middle, scale bar: 500 nm) and confocal microscopy images (right, scale bar: 1 μm) illustrating ICG-conjugated ClyA-ARG bacteria (CAB@ICG, upper) or ICG-conjugated ClyA-GVs bacteria (CGB@ICG, below). (C) Zeta potential measurements of ICG, CGB, and CGB@ICG in PBS solution. (D, E) UV-vis spectra of ICG and CGB@ICG at different concentrations, with an excitation wavelength at 810 nm related to ICG. (G) Temperature profiles of ICG, CGB, and CGB@ICG dispersions at different time points under 808 nm irradiation for 5 minutes (1 W/cm^2^, 50 μg/mL ICG attached to 5×10^8^ CFU/mL CGB). (H) Temperature elevation curves of 5×10^8^ CFU/mL CGB@ICG as a function of ICG concentrations under 808 nm irradiation (1 W/cm^2^) and 50 μg/mL ICG attached to 5×10^8^ CFU/mL CGB across (I)various power intensities. (J) Temperature changes in CGB@ICG cultures (5×10^8^ CFU/mL, 50 μg/mL ICG) through three ON/OFF cycles (808 nm, 0.75 W/cm^2^). SDS-PAGE analysis of (K, N) temperature-dependent ClyA protein expression (red arrow) in CGB@ICG and (L, O) time-dependent expression of ClyA protein in CGB@ICG at 42 °C. (M)Temperature elevation curves of CGB@ICG as a function of bacterial concentrations under HIFU irradiation.

An image-guided, thermally controlled bacterium (CGB@ICG) consisting of engineered bacteria and ICG was developed in this study. Fig. 2A presents the images of three distinct bacterial strains, illustrating a white or green meniscus layer floating on the surface of PBS solutions. The CAB group did not produce a white or green meniscus, highlighting the presence of GVs in both the CGB group and CGB@ICG group. These GVs are protein-encased nanostructures filled with air, designed to achieve cellular buoyancy^41^. A considerable number of cylindrical- or spindle-shaped^42^ GVs were observed, occupying a significant portion of the intracellular volume (Fig. 2B). These findings demonstrate the expression of GVs induced by IPTG and arabinose in bacteria. Fluorescence imaging revealed that ICG was located on the surface of CGB, facilitating the conjugation between CGB and ICG (Fig. 2B). The zeta potentials of ICG and CGB in PBS were approximately -4.87 ± 0.85 mV and -9.88 ± 1.02 mV, respectively. At a pH greater than 2, most bacteria exhibited a negative zeta potential, attributed to negatively charged functional groups on their surface^43^. Following the combination of CGB and ICG, the zeta potential was measured at -21.33 ± 1.65 mV, indicating the formation of a stable, negatively charged amide bond between CGB and ICG (Fig. 2C). Subsequent analysis revealed that the UV spectra of CGB@ICG exhibited a high absorption range (800-820 nm), comparable to that of ICG and distinct from CGB (Fig. 2D). Furthermore, the absorption peak value of CGB@ICG was directly proportional to the ICG concentration (Fig. 2E). The efficiency of bacterial adhesion to ICG was found to be over 50%, as determined by evaluating the binding efficiency of ICG at various concentrations on the surface of CGB (Fig. 2F). These results confirmed the successful creation of CGB@ICG bacteria, which exhibited fluorescence characteristics identical to those of ICG.

Given that ICG strongly absorbs NIR wavelengths to generate heat^44^, it was hypothesized that CGB@ICG possesses superior photothermal conversion properties. Compared to CGB@ICG, ICG alone generated a slightly lower temperature at the equivalent concentrations, likely attributable to the higher specific heat capacity of CGB@ICG (Fig. S1). Subsequent experiments irradiated different ICG concentrations within CGB@ICG groups with NIR, revealing that increased ICG concentrations in CGB@ICG correlated with faster heating rate and a higher final temperature (Fig. 2G). Temperature changes in response to the same irradiation conditions were also measured across varying bacterial concentrations (10^8^-10^9^ colony forming units (CFU)/mL), demonstrating a positive correlation between bacterial concentration and temperature increase (Fig. 2H). Furthermore, temperature trends of CGB@ICG-treated bacteria under varying laser power densities (0.5-1.5 W/cm^2^) are depicted in Fig. 2I. As anticipated, a time-dependent increase in both temperature and heating rate was observed following the enhancement of laser power density, indicating that the photothermal heating effect of CGB@ICG-treated bacteria could be precisely modulated by altering experimental conditions. Notably, under the conditions of 50 μg/ml ICG, 5×10^8^ CFU/ml, and 1 W/cm^2^, the temperature reached 42 °C within approximately 1 minute and continued to rise above 42 °C by with sustained NIR laser irradiation. Additionally, the photothermal conversion stability of CGB@ICG was further evaluated by measuring the cyclic photothermal properties through repeated on-and-off laser irradiation cycles. CGB@ICG exhibited a lower temperature variation after three cycles of laser irradiation (Fig. 2J). Besides, the temperature curves of CGB@ICG in different bacterial concentrations irradiated by HIFU was observed. As shown in Fig. 1M, the temperature increase was observed following the enhancement of CGB@ICG concentrations. These findings underscore the exceptional photothermal properties of CGB@ICG, establishing a foundation for thermally induced protein expression.

To further investigate the thermal sensitivity of CGB@ICG, the thermally activated expression of ClyA protein at various temperatures was assessed using SDS-PAGE analysis. As depicted in Fig. 2K, N, the expression of ClyA protein significantly varied with the increase in culture temperature, sampled from the centrifugal precipitation of CGB@ICG after a 30-minute incubation at the specified ambient temperature. At 30 °C, ClyA expression was negligible, yet it became detectable at 42 °C, thereby confirming the thermal sensitivity of CGB@ICG. Additionally, the expression of ClyA protein at 42 °C was found to be time-dependent, as demonstrated by sampling at designated culture time points (Fig. 2L, O). ClyA expression was discernible after a 10-minute laser irradiation, with further culture time amplifying ClyA expression. Notably, ClyA expression was observed after a 20-minute HIFU irradiation. Consequently, it was determined that the expression of ClyA protein is precisely regulated by both ambient temperature and culture duration via acoustic- or photothermal effects.

### *In vitro* and *in vivo* multimodal imaging properties of CGB@ICG bacteria

**Figure 3:**
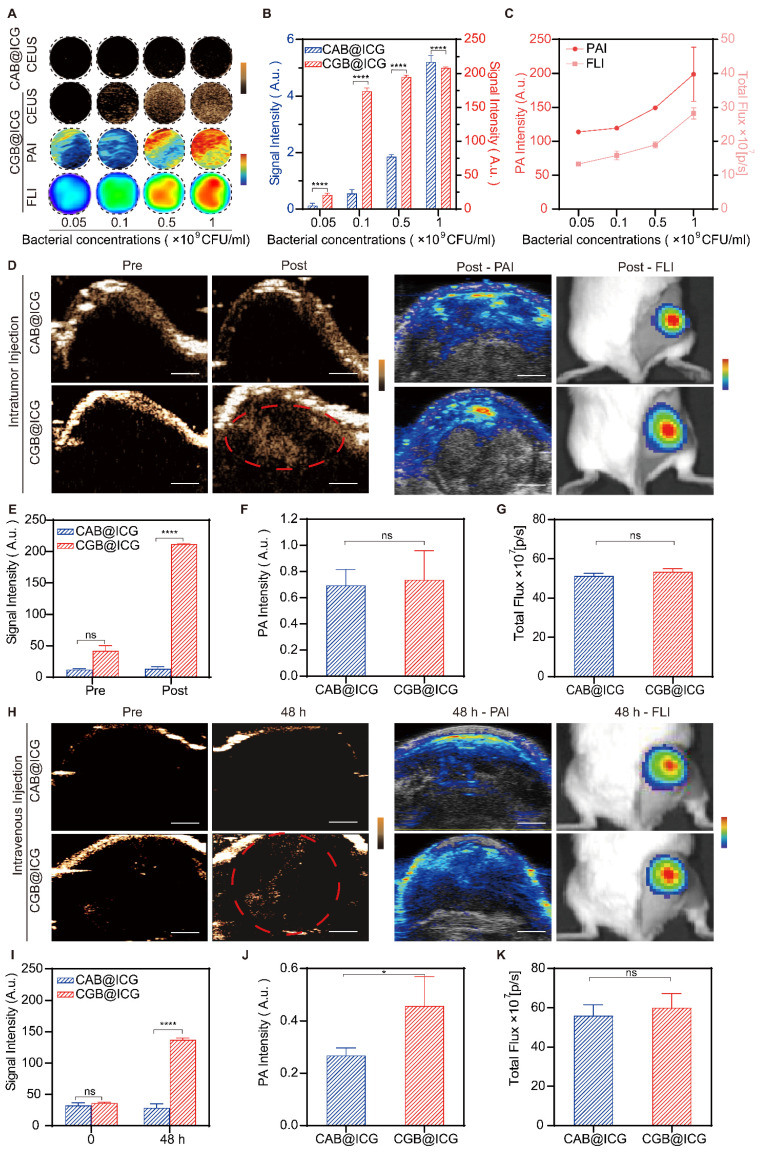
*In vitro* and *in vivo* multimodal imaging of CGB@ICG. (A) Contrast-enhanced ultrasound/photoacoustic/fluorescence images were captured in a concentration series of CAB@ICG and CGB@ICG bacteria. (B) Quantitative analysis of ultrasound signal intensities from CAB@ICG and CGB@ICG bacteria, and (C) the photoacoustic intensities and total flux intensities of CGB@ICG at different bacterial concentrations. (D) Contrast-enhanced ultrasound/photoacoustic/fluorescence images of CAB@ICG and CGB@ICG before and after intratumoral injection of bacteria at a concentration of 10^9^ CFU/mL. Scale bar, 1 mm. (E, F, G) Quantitative analysis of the ultrasound signal, photoacoustic intensities, and total flux intensities before and after intratumoral injection. (H) Contrast-enhanced ultrasound/photoacoustic/fluorescence images of CAB@ICG and CGB@ICG bacteria before and after intravenous injection of 2×10^8^ CFU/ml bacteria at various time points. Scale bar, 1 mm. (I, J, K) Quantitative analysis of the ultrasound signal, photoacoustic intensities, and total flux intensities before and after intravenous injection at various time points. Data are presented as mean ± SD (n=3, ^*^p< 0.05, ^****^p< 0.0001), and two-way ANOVA was performed using GraphPad Prism.

Previous studies have demonstrated the outstanding contrast-enhanced ultrasound imaging capabilities attributable to the presence of GVs in bacteria^37^, ^45^. To ascertain the imaging threshold value of CGB@ICG under induction conditions, CGB@ICG was subjected to a series of IPTG concentrations and discovered that ultrasound signals were generated at concentrations as low as 0.4 mM IPTG (Fig. S2). In an effort to evaluate whether the ultrasound signals emitted by CGB@ICG could be modulated by the concentration of bacterial cells, CGB@ICG at concentrations from 5×10^7^ to 10^9^ CFU/mL was examined, with CAB@ICG serving as the control group. As depicted in Fig. 3A, to further elucidate the *in vitro* multimodal imaging capabilities of CGB@ICG, CGB@ICG was observed to produce distinct ultrasound contrast signals in comparison to CAB@ICG, and the signal intensities of CGB@ICG in the PAI and FLI experiments were amplified with increasing bacterial concentrations. Remarkably, GVs enriched in CGB@ICG yielded a superior contrast signal relative to the control group, underscoring the enhanced ultrasound imaging sensitivity of CGB@ICG facilitated by GVs^46^ (Fig. 3B). Quantitative analysis indicated that the PA value and total flux increased linearly with the concentration of CGB@ICG (Fig. 3C). Furthermore, we firstly observed the* in vivo* imaging capacity of CGB@ICG pre- and post-intratumoral injection. Tumor tissues inoculated with CAB@ICG bacteria exhibited no signal in contrast ultrasound imaging. Conversely, tumor tissues treated with CGB@ICG bacteria displayed a pronounced enhancement of the signal post-injection, suggesting that CGB@ICG affords a heightened sensitivity of ultrasound imaging *in vivo* compared to CAB@ICG (Fig. 3D, E). Given the equivalent binding efficiency of ICG attached to the bacteria administered intratumorally^47^, it is imperative to acknowledge that no significant disparity in signal was observed between the two groups in PAI and FLI experiments (Fig. 3F, G). These findings confirmed that CGB@ICG possessed capabilities for ultrasound, photoacoustic, and fluorescence multimodal imaging *in vivo*. Thus, we hypothesized that the multimodal imaging capacity of CGB@ICG enabled the *in vivo* tracing of bacterial locations, which may offer a solution to mitigate the limitations of existing monitoring methodologies, providing precise locations of bacteria* in vivo* 48.

To date, the intravenous injection of bacteria has been exhibited outstanding antitumor effect while the concern about* in vivo* biodistribution is still existing. Hence, real-time imaging of bacteria plays significant role to improve biosafety within intravenous injection of bacteria. In this study, subsequent imaging of tumor tissues post-intravenous injection with either CAB@ICG or CGB@ICG bacteria were detected at various time point for precisely monitoring the accumulation of bacteria in tumor. The results revealed that, relative to the CAB@ICG group, the ultrasound images of tumors in the CGB@ICG group exhibited a significant amplification in signal 48 hours post-injection (3.86-fold). The ultrasound signal intensity in the CGB@ICG group was 5.56 times stronger than that in the CAB@ICG group (Fig. 3H, I), aligning with prior results. The photoacoustic intensities displayed a significant variance between the two groups 48 hours post-injection. Nevertheless, no marked difference in fluorescence signal was observed between the groups (Fig. 3J, K). These outcomes demonstrate that CGB@ICG serves as an efficacious imaging agent that enhances imaging performance. The complementary imaging characteristics of these three techniques imply that multimodal imaging employing CGB@ICG can be effectively utilized for monitoring purposes.

### *In vitro* synergistic cytotoxicity of ClyA protein and PTT

To demonstrate the superiority of CGB@ICG compared to other *E. coli* strains, *in vitro* experiment was designed for evaluating *in vitro* cytotoxicity of ClyA protein which was expressed by *E. coli* BL21 (CGB@ICG) or *E. coli* MG1655 (CGM@ICG). Five monoclonal colonies of each selected bacteria strains were randomly selected, amplified, and incubated with cells. Then the co-cultures were subject to irradiation and the temperature maintained at 42 °C. The result demonstrated that the CGB@ICG groups exhibited a stronger cytotoxicity than that of CGM@ICG groups (Fig. S3).

Previous studies have demonstrated that HIFU can induce gene expression from a thermally inducible system 27. Additionally, the attachment of ICG to the surface of CGB can facilitate the conversion of NIR laser (L) irradiation into heat, thereby inducing ClyA protein expression. However, it is usually difficult to achieve satisfactory results with bacterial monotherapy alone. Thus, combination therapy has emerged as an effective strategy against tumors. Encouraged by these findings, the cytotoxicity of ClyA protein expressed by CGB@ICG induced by these two methods was assessed, with a second NIR laser irradiation provided for photothermal therapy. Initially, bacterial activity on agar plates at different temperatures was evaluated. Colonies treated at 42 °C for 20 minutes showed no significant difference compared to those treated at 37 °C for 20 minutes (Control group). However, no colonies grew after treatment at 50 °C for 10 minutes (Fig. S4), indicating that colonies at 42 °C have no significant effect on bacterial survival, while 50 °C irradiation can eliminate bacteria after 10 minutes. Thus, the second laser irradiation can eliminate any remaining bacteria in the tumor, in addition to enhancing the photothermal therapy effect to improve safety.

**Figure 4:**
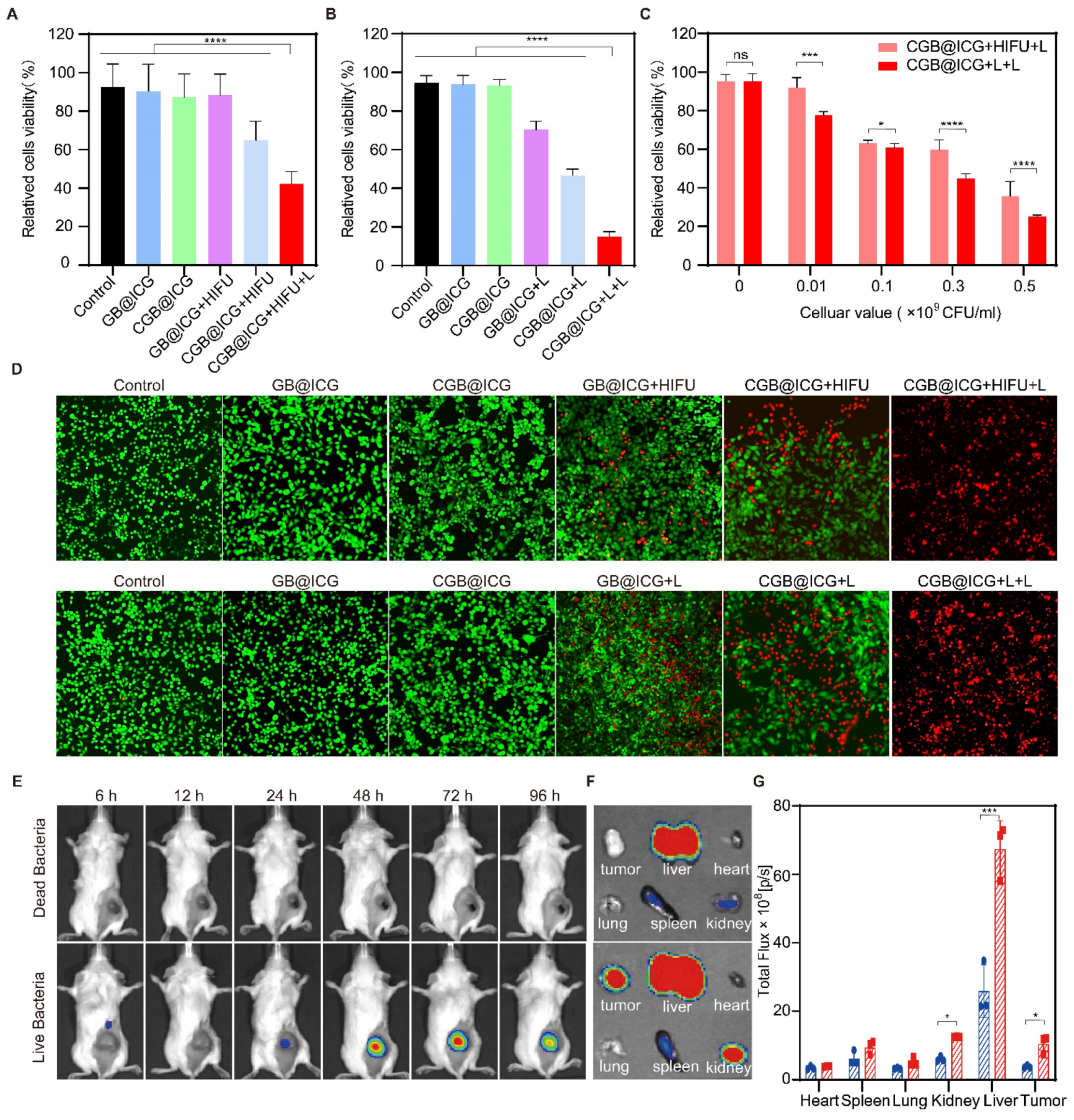
* In vitro* synergistic cytotoxicity and* in vivo* biodistribution of CGB@ICG. (A, B) Viability of 4T1 cells *in vitro* after co-incubation with CGB@ICG or GB@ICG, with or without induced ClyA protein expression (808 nm, 42 °C, 20 minutes) by high-intensity focused ultrasound (HIFU) or Laser (L), and with or without subsequent laser irradiation (808 nm, 55 °C, 10 min). (C) Relative viability difference between CGB@ICG+HIFU+L and CGB@ICG+L+L at various cellular concentrations. (D) Live and dead double-staining images of 4T1 cells co-incubated with different therapeutic approaches. (E) *In vivo* fluorescence imaging of tumor-bearing mice at different times after intravenous injection of 2×10^8^ CFU of live CGB@ICG or heat-inactivated dead CGB@ICG (n = 6 per group). (F) Fluorescence image of isolated tissues at 48 hours post-injection and (G) corresponding fluorescence intensities of isolated organs and tumors. Data are presented as mean ± S.D. Statistical analysis was performed by using a one-way analysis of variance with Tukey's test (^*^p< 0.05, ^***^p< 0.001, ^****^p< 0.0001).

Cytotoxicity resulting from exposure of ClyA protein or PTT was evaluated using a CCK-8 kit assay. As depicted in Fig. 4A, CGB@ICG+HIFU and GB@ICG+HIFU groups were established to assess cancer cell death caused by ClyA protein or HIFU, respectively. Neither GB@ICG nor CGB@ICG affected the viability of 4T1 tumor cells, indicating their safety. Subsequently, cell viability was 35.11% ± 18.18%, 64.81% ± 8.99%, and 88.2% ± 9.90% for CGB@ICG+HIFU+L, CGB@ICG+HIFU, and GB@ICG+HIFU, respectively. The results suggested that ClyA protein or PTT affected tumor cells compared to the control group, with the CGB@ICG+HIFU+L group, receiving combination therapy of ClyA protein and PTT, demonstrating enhanced damage to tumor cells.

Next, ClyA protein expression was induced using the NIR laser. The cancer cell viability was 15.05% ± 2.19% for the CGB@ICG+L+L group, significantly lower than that of any therapeutic treatment (Fig. 4B). The efficacy of combining ClyA protein induced by different approaches with PTT was assessed at bacteria cell concentrations ranging from 1×10^7^ to 5×10^8^ CFU/mL. Cancer cell viability decreased with increasing concentration of bacteria cells, indicating synergistic efficacy of ClyA protein induced with the NIR laser compared to induction from HIFU (Fig. 4C).

To measure cell viability from combination therapy and single therapeutic approaches, live (green) and dead (red) cells were imaged by fluorescence imaging. The images also demonstrated that cancer cell death caused by ClyA protein combined with PTT was higher than that resulting from any therapeutic approach (Fig. 4D). In summary, these results highlight the potential synergistic effect of ClyA protein induction with PTT in inhibiting tumor growth.

### Tumor targeting with CGB@ICG

Because therapeutic efficacy depends on the concentration of therapeutic protein expressed from bacteria, it is necessary to confirm inducible timepoint of maximum bacterial concentration in tumor 49. We have proved that the multimodal imaging ability of CGB@ICG after intratumoral injection and intravenous injection. It was reported that intratumoral injection can directly deposit bacteria within target tumors, which can reach maximum bacterial concentration compared to intravenous injection 50. Thus, we developed tumor-targeting experiments to confirm the inducible timepoint of CGB@ICG to express ClyA protein in tumor after intravenous injection.

Dead bacteria group lost the ability to invading, colonizing solid tumors and self-propulsion of penetrating tumors after treatment at 70 °C for 2 hours, which can be used as a control group. Leveraging the high fluorescence imaging capability of ICG, the *in vivo* accumulation of CGB@ICG were able to be directly observed without external modification. Live or dead CGB@ICG bacteria were intravenously administered to 4T1 tumor-bearing mice, followed by imaging at various time points. As depicted in Fig. 4E, a robust fluorescence signal was evident at the tumor sites 24 hours post-injection. The fluorescence intensity at these sites continued to increase over time, plateauing after 48 hours. Notably, visible fluorescence signals persisted in tumor sites even at 96 hours post-injection, indicating the prolonged presence of CGB@ICG at the tumor site. Conversely, fluorescence intensity from dead CGB@ICG bacteria was not observed in the tumor sites. A quantitative analysis of the fluorescence intensity at different time points further underscored the tumor-targeting capability of CGB@ICG (Fig. S5).

To gain deeper insights into bacterial accumulation at tumor sites, major organs (heart, liver, spleen, lung, and kidney) and tumor tissues were isolated 48 hours post-injection (Fig. 4F). It was observed that fluorescence signals from dead CGB@ICG predominantly concentrated in the liver, with some accumulation in the spleen and kidney. In contrast, the live CGB@ICG group exhibited higher accumulation in both tumor sites and organs compared to the dead CGB@ICG group. Considering that the liver and spleen are the initial organs for localization following intravenous administration, these findings were anticipated. Quantitative analysis revealed a 2.23-fold higher signal in tumor sites from the live CGB@ICG bacteria compared to the dead group (Fig. 4G).

Subsequently, organs and tumor tissues were isolated, homogenized, and coated onto LB plates, followed by culture at 37 °C for 12 hours. Live CGB@ICG demonstrated concentration in the liver and tumor tissues, as evidenced by colony growth from these tissue lysates, while dead CGB@ICG did not yield colonies on the plates (Fig. S6). This observation can be attributed to the loss of activity in dead bacteria treated with high temperatures. Overall, our findings elucidate the tumor-targeting capacity of CGB@ICG after intravenous injection, highlighting its potential as a delivery vector* in vivo*.

### Enhancement of* in vivo* PTT in tumor treatment through focused ultrasound-induced ClyA expression

**Figure 5:**
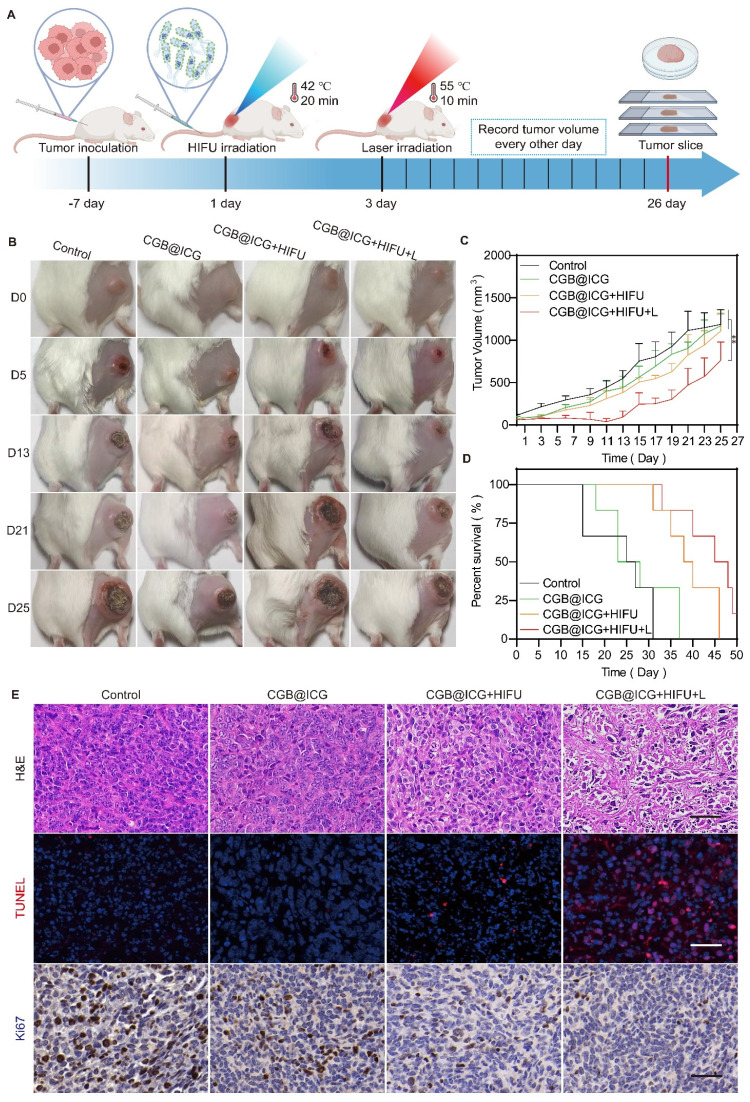
Synergistic antitumor effect of ultrasound-induced ClyA expression and photothermal therapy. (A) Schematic diagram illustrating the experimental procedure of CGB@ICG-mediated combination therapy in tumor-bearing mice. (B) Representative images of tumor-bearing mice after receiving injections of PBS, CGB@ICG, CGB@ICG + HIFU, or CGB@ICG + HIFU + L injections. (C) Tumor volume curves during therapy with different treatments. (D) Survival curves of tumor-bearing mice undergoing different treatments. (E) H&E, TUNEL, and Ki67 staining of tumor tissues. Scale bar =50 μm. Images are representative of three independent experiments. Data are presented as mean ± S.D. Statistical analyses were performed using a two-way analysis of variance with Tukey's test (^**^p< 0.001).

Prior to *in vivo* treatment, the photothermal imaging properties of CGB@ICG in tumors were first investigated to confirm tumor temperature using NIR laser irradiation. Compared with the CGB group, the temperature of CGB@ICG tumors increased by 10.7 °C in one minute and reached 54.2 °C within five minutes (Fig. S7). These results demonstrate that CGB@ICG possesses outstanding photothermal properties in the tumor and has the potential for tumor ablation. To evaluate the synergistic antitumor effect of focused ultrasound-induced ClyA protein expression combined with PTT *in vivo*, 4T1 tumor-bearing mice were treated following the therapeutic schedule outlined in Fig. 5A. Mice were randomly separated into four groups and treated with PBS (control), CGB@ICG without ClyA protein expression (CGB@ICG), ClyA protein expression induced by focused ultrasound (CGB@ICG+HIFU), or a combination of ClyA protein and NIR laser irradiation (CGB@ICG+HIFU+L). Forty-eight hours post-intravenous injection, focused ultrasound was used to irradiate the tumor to trigger the expression of ClyA protein. Tumor growth was considerably inhibited in the CGB@ICG+HIFU+L group compared with the other groups (Fig. 5B, C). It should be noted that there was no significant difference in baseline tumor volume between the groups. A slight antitumor effect of CGB@ICG+HIFU could be observed, which could be attributed to ClyA protein expression. Furthermore, the mean survival time increased by 45 days in the CGB@ICG+HIFU+L group compared with 25 days in the control group (Fig. 5D). Body weight increased slightly in all the groups (Fig. S8A). These results show that the synergistic therapy of ClyA protein and PTT had a strong inhibitory effect on tumor growth. Hematoxylin and eosin (H&E) and TdT-mediated dUTP nick-end labeling (TUNEL) staining of the tumor tissues were conducted after the different treatments to measure the synergistic effect of ClyA protein and PTT (Fig. 5E). Based on the H&E images, there was no obvious change in cell morphology in the control group and CGB@ICG group, while H&E staining of the CGB@ICG+HIFU+L group showed a slight separation between the cell nucleus and cytoplasm. The results indicate that the tumor cells were undergoing apoptosis, confirming the synergistic effect of ClyA protein and PTT produced by the CGB@ICG. Using the TUNEL assay, red staining was observed in tissue from the CGB@ICG+HIFU+L group, indicating apoptotic cells. Ki67 staining revealed the least number of proliferating cells in the ClyA protein and PTT groups. These results reveal that this combination therapy could regulate the cell cycle and suppress tumor growth.

### *In vivo* enhancement of tumor PTT through NIR laser-induced ClyA expression

**Figure 6:**
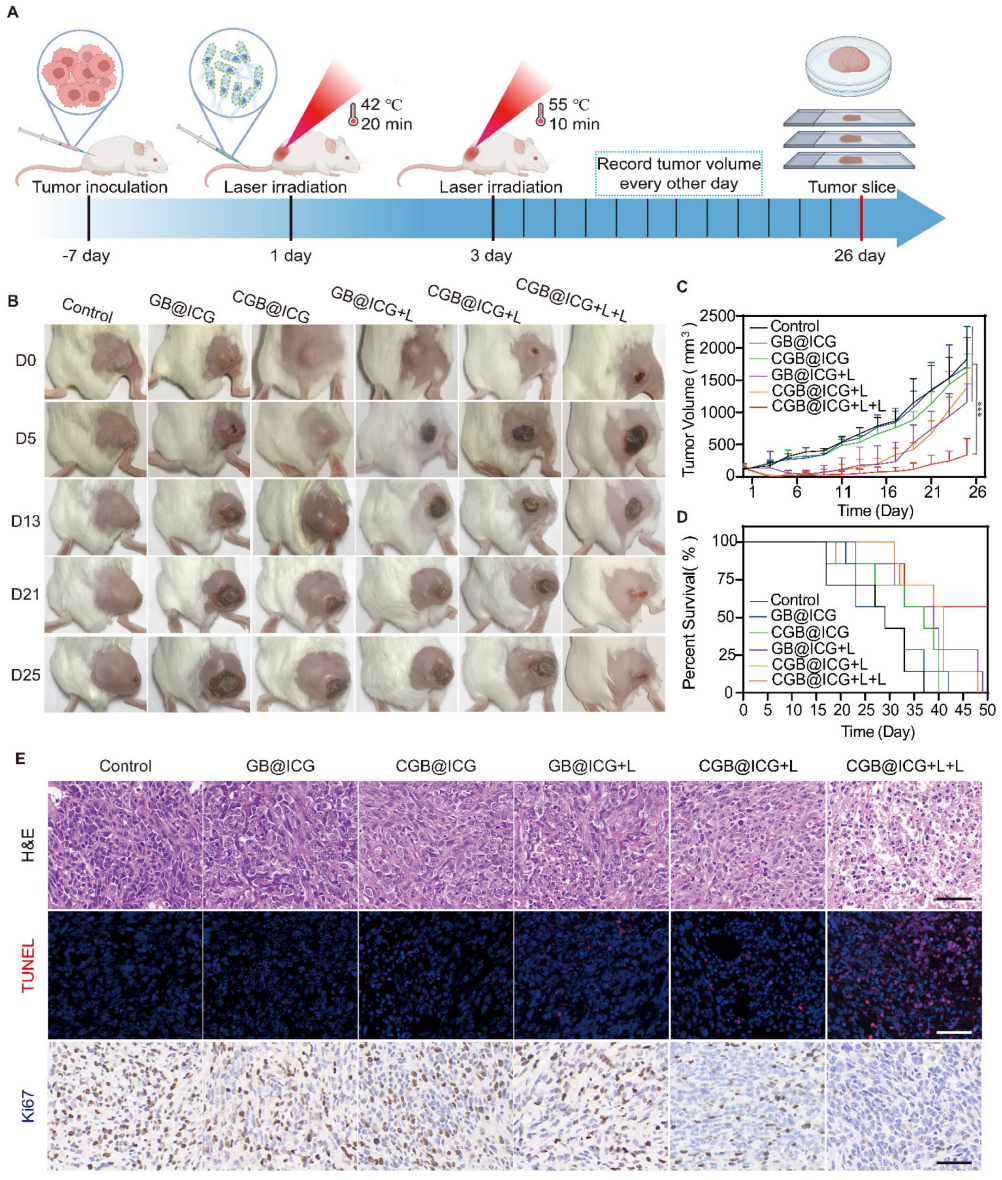
Synergistic effect of NIR laser-induced ClyA expression and photothermal therapy of tumors. (A) Schematic diagram illustrating the experimental procedure of CGB@ICG-mediated combination therapy in tumor-bearing mice. (B) Representative images of tumor-bearing mice treated with PBS, GB@ICG, CGB@ICG, GB@ICG+L, CGB@ICG+L, or CGB@ICG+L+L. (C) Tumor volume curves during treatment with different therapies. (D) Survival curves of tumor-bearing mice undergoing different treatments. (E) H&E, TUNEL and Ki67 staining of tumor tissue slides from each group. Scale bar =50 μm. Images are representative of three independent experiments. Data are presented as mean ± S.D. Statistical analyses were performed using a two-way analysis of variance with Tukey's test (^***^p< 0.001).

As depicted in Fig. 4, the synergistic therapeutic effect of HIFU and PTT surpassed that of Laser and PTT. This is primarily because the area irradiated by NIR is larger than that irradiated by HIFU, thus allowing CGB@ICG to express more ClyA protein. Importantly, the ultrasound energy decrease with increasing tumor depth, thereby resulting in insufficient ultrasound energy deposition in deep and large tumor tissues 51. Thus, it is challenges to improve anti-tumor effect without surrounding tissues damage. Previous studies have reported that ICG can absorb NIR laser irradiation and convert it into heat to induce thermal toxicity^52^. Therefore, we intended to use the stable photothermal properties of ICG to control the tumor temperature up to 42 °C and stimulate the expression of ClyA protein. Subsequently, the NIR laser was applied again to irradiate tumor sites to reach 50 °C for 10 minutes to eliminate residual bacteria in the tumor and enhance tumor cell killing (Fig. 6A). Mice were divided into six groups to receive different treatments: PBS, GB@ICG, CGB@ICG, GB@ICG+L, CGB@ICG+L, and CGB@ICG+L+L. The GB@ICG and GB@ICG+L groups were used to evaluate the antitumor effect of the single therapeutic approach of PTT. During treatment, body weight and tumor volume were recorded every other day. The body weights of mice in each group were similar during treatment (Fig. S8B). Compared with the PBS control group, the tumor volume of the CGB@ICG+L+L group did not significantly increase after treatment. Digital images of all tumor tissues were acquired, revealing rapid tumor growth in the PBS group (Fig. 6B). Tumor growth in the GB@ICG+L and CGB@ICG+L groups was slightly inhibited, attributable to the cytotoxic effect of PTT or ClyA protein, respectively. CGB@ICG+L+L substantially inhibited tumor growth (400 mm^3^) compared to the PBS group (1,800 mm^3^). Moreover, the therapeutic effect of CGB@ICG+L+L was superior to that seen in the GB@ICG+L and CGB@ICG+L groups (Fig. 6C). This combined benefit was attributed to the synergistic effect of ClyA expression and PTT. Notably, the survival rate of mice in the CGB@ICG+L+L group was higher than that of the control group (Fig. 6D). These data indicate that CGB@ICG+L+L significantly inhibited tumor growth and improved the survival of tumor-bearing mice.

At the end of the study, mice were euthanized to allow for the isolation of organs and tumor tissues for H&E and TUNEL staining. Numerous nuclear condensation events and cell shrinkage were evident in the H&E staining after treatment with CGB@ICG+L+L, indicating severe tumor cell damage (Fig. 6E). Similarly, the TUNEL assay demonstrated high cell apoptosis (red) in this group. Ki67 staining revealed the least amount of proliferating cells in the ClyA protein and PTT groups with a positivity rate of 10.28%, compared to 62.66% in the control group. These findings support the improved therapeutic efficacy of mice treated with CGB@ICG+L+L. The observed difference compared to mice treated with GB@ICG+L and CGB@ICG+L were also explained by the synergistic effect of ClyA protein and PTT.

There is a desire to assess* in vivo* biosafety of bacteria-based tumor therapy. Thus, the biosafety of CGB@ICG with different therapeutic treatments was assessed from various aspects. Blood samples collected after 1 day and 14 days of injection were analyzed for inflammatory responses and liver and kidney function indicators. Routine blood tests showed no obvious change at the two sampling time points (Fig. S9). Additionally, liver function-related indicators such as ALT and AST, and kidney function-related indicators like UREA and CREA, fluctuated within the normal level range compared to the control group (Fig. S10). Simultaneously, H&E staining revealed no obvious histological, morphological, and pathological changes in a range of organs (heart, liver, spleen, lung, kidney) after injection of bacteria with different treatments (Fig. S11). These results underscore the potential application of combined biotherapy and PTT in tumor therapy without adverse effects.

## Discussion

The utilization of gene-expressing bacteria for therapeutic protein-releasing in tumor represents a promising methodology for tumor therapy, while the routes of bacteria administration are crucial for anti-tumor efficacy 53. Intratumoral injection offers the advantages of the maximum bacterial concentration in tumor and the minimum systemic toxicity 54. Yet it is notable that abnormal pressure in tumor tissue results in insufficient penetration and diffusion, which is not preferable for the treatment of deep-seated tumors 55. Compared to intratumoral injection, it was reported that intravenous injection of gene-expressing bacteria has a remarkably anti-tumor efficacy 56, 57. This may primarily because the bacteria can diffusely distribute in tumor through the abundant tumor vessels for expanding the treatment area. However, A critical concern about route of intravenous injection is to take into consideration the balance of effective and safe dosage of bacteria. To address the concern, one strategy is to dynamically control the expression of therapeutic protein through genetically manipulate bacteria, while the premise is to visualize the gene-expressing bacteria for confirming the inducible timing 58. Additionally, since the anti-tumor efficiency of bacterial monotherapy is not prominent, synergistic gene-expressing bacteria-mediated photothermal therapy represents an appealing method for tumor therapy 59-61. In this study, we aimed to develop US/FL/PA multimodal imaged gene-expressing bacteria (named CGB@ICG) by combining the acoustic reporter gene strategy with the surface marker strategy. The multimodal imaging-guided gene-expressing bacteria could i) decrease the background uptake of the normal bacterial strain to evaluate the accumulation of bacteria in tumor, ii) monitor the accuracy of delivery to the tumor* in vivo* to confirm the inducible timepoint, and iii) enhance anti-tumor effects by combining the ClyA protein therapy and PTT therapy. Hence, multimodal imaging-guided gene-expressing bacteria represents a promising model for guiding follow-up combination therapy.

BMTT has overcome the limitations of conventional cancer treatments because of effective accumulation and penetration of bacteria into tumor for the diagnosis and treatment of tumor. However, the biosafety has become a prominent issue in BMTT. As a result, few BMTT has reached the clinical trial stage. This may be because accurately localizing the bacteria at different sites is of great importance. However, existing BMTT in clinical trials use subjective clinical signs and invasive sampling to confirm the localization of bacteria. Hence, it is necessary to utilize non-invasive and repetitive* in vivo* imaging techniques to enhance the visualization of bacteria *in vivo*, thereby facilitating the clinical transformation of BMTT. Currently, *in vivo* optical imaging techniques are extensively employed to image the bacteria in most preclinical studies, while the application of *in vivo* optical imaging for clinical studies is limited due to the low penetration capacity 45-46. Therefore, the development of novel imaging techniques is imperative to image bacteria for improving biosafety.

Multimodal imaging with the combination of two or more imaging modes can combine the advantages of different imaging methods and complement their shortcomings to provide high spatial resolution anatomical information of the disease site, highly sensitive biological information at the molecular level and time resolution scales. While fluorescence and bioluminescence optical imaging have been extensively employed due to their high sensitivity, they suffer from certain limitations in achieving specific imaging. Despite the development of near-infrared fluorophores to mitigate light absorption and enhance penetration depth, discriminating between normal and therapeutic bacteria *in vivo* remains challenging. Additionally, previous studies have introduced various bioluminescence reporter genes for *in vivo* bacterial imaging, but the signal may diminish over time 34. Moreover, concerns regarding safety arise from the requirement of exogenous substrates for bioluminescence reporter genes. Recently, acoustic reporter genes have emerged as an alternative approach for specific bacterial imaging, showing promising results 33. These genes entail heterologous multigene clusters encoding GVs that are incorporated into bacterial strains like *E. coli* and *S.typhimurium* to express GVs, enabling visualization through ultrasound imaging. Overall, optical imaging is unable to provide supplementary anatomical or physiological data, nor can they offer the highly resolution for non-invasive analysis of the efficacy. As mentioned above, CEUS has also been explored to provide the localization of bacteria *in vivo* microvascular, while the application of CEUS is limited in sensitivity. PAI represents a promising solution with the combination of high resolution and deep penetration. However, due to the tumor heterogeneity, the complexity of light absorption and scattering may cause light attenuation in tumor. Thus, we constructed a multimodal imaging bacterium. In our study, near-infrared fluorophores were attached to the surface of these engineered bacteria containing GVs to achieve multimodal imaging of therapeutic bacteria. These near-infrared fluorophores facilitate fluorescence imaging and photoacoustic imaging, while GVs enable contrast-enhanced ultrasound imaging. The concentration of therapeutic bacteria is a crucial factor to consider, with significant contrast signals observed even at low bacterial concentration of 5 × 10^8^ CFU/ml, providing sufficient sensitivity for *in vivo* imaging 35. Upon intertumoral injection of therapeutic bacteria, contrast signals were predominantly concentrated in the central region of the tumor in contrast-enhanced ultrasound imaging (CEUS). In photoacoustic imaging (PAI), the signal of therapeutic bacteria was observed in the upper part of the tumor, while in fluorescent imaging (FLI), it was distributed throughout the tumor. Notably, the accurate localization of therapeutic bacteria was more evident in CEUS compared to FLI due to the superior penetration performance of ultrasound. Additionally, the disparity observed between PAI and CEUS is primarily attributed to the presence of gas vesicles in therapeutic bacteria, enhancing the signal in CEUS by 2.4-fold according to second-harmonic imaging. Our results demonstrate that multimodal imaging enables precise localization of therapeutic bacteria within tumors. Furthermore, by monitoring the delivery of therapeutic bacteria after intravenous injection at different time points, we observed that the contrast signal peaked at 48 hours post-injection, remaining concentrated in the deep region of the tumor. While photoacoustic signals were detectable in the deep region of the tumor, the area of photoacoustic signal was larger than that of the contrast signal, possibly due to the penetration of free near-infrared fluorophores into the tumor tissue via the enhanced permeability and retention effect, leading to non-specific bacterial imaging. Overall, *in vivo* data from this study corroborate the precise monitoring and localization capabilities of multimodal imaged therapeutic bacteria.

It is notably that the combination of multimodal imaging and treatment has been attracted growing interest. A number of multimodal therapeutic probes have been developed to guide tumor therapy through multimodal imaging for improving antitumor efficacy. Specific bacterial imaging plays a pivotal role in monitoring and confirming the accurate delivery of therapeutic bacteria to target lesions, thereby enabling precise determination of the trigger time for gene expression. In our study, the maximum accumulation of therapeutic bacteria in tumor was observed 48 hours after injection, with gene expression subsequently triggered by either HIFU or laser to induce ClyA protein expression. Previous investigations have highlighted that ClyA protein released by bacteria can arrest the cell cycle 30. In line with this, earlier research has shown that the tumor cell inhibition rate attributed to ClyA protein expression by bacteria under laser induction was 22% at bacterial concentrations of 2×10^8^ CFU/ml 28. Interestingly, in our study, a significantly higher suppression percentage of tumor cells was observed at a lower bacterial concentration of 1×10^8^ CFU/ml. This discrepancy led us to consider potential mechanisms underlying these observations. We hypothesized that therapeutic bacteria containing gas vesicles might experience simultaneous bursting during HIFU or laser induction, resulting in a weaker sonoporation effect. Furthermore, the therapeutic effect induced by HIFU group was noted to be less effective compared to that induced by laser. This disparity could be attributed to the smaller irradiated area achieved with HIFU, potentially leading some therapeutic bacteria failing to express therapeutic proteins. Moreover, temperature stability in the laser-induced group was observed to be superior to that in the HIFU-induced group, suggesting that some bacteria may not have been irradiated for the intended duration. Taken together, these findings underscore the potential applicability of therapeutic protein expressed from imaged therapeutic bacteria for tumor treatment. They also highlight the importance of optimizing the delivery and induction methods to maximize therapeutic efficacy while minimizing potential limitations associated with specific induction techniques.

The efficacy of bacterial monotherapy in tumor treatment has been found to be unsatisfactory 62. Therefore, synergistic therapy has emerged as a promising approach to achieve stronger therapeutic outcomes compared to bacterial monotherapy 63. Conventional photothermal therapy (PTT) often faces challenges such as inadequate intertumoral accumulation and insufficient penetration of photothermal agents (PTA) due to tumor heterogeneity and the high interstitial pressure of the tumor microenvironment, leading to incomplete tumor eradication 64-66. Various targeting strategies, including passive and active targeting approaches, have been developed extensively to improve the selective accumulation of PTAs in tumors and enhance the therapeutic efficacy of PTT 67, 68. However, passive strategies may not entirely eliminate the accumulation of photothermal agents in tumor-adjacent tissues and organs, while the effectiveness of active targeting is limited by poor tumor penetration. In this context, leveraging bacteria as carriers to deliver photothermal agents offers a promising solution to address these challenges. Imaged therapeutic bacteria, serving as a drug delivery platform, possess unique tumor-targeting capabilities when combined with photothermal agents. Therefore, the combination therapeutic effect of ClyA protein with PTT was further assessed. Our results demonstrated that the combination therapy group exhibited a rapid decrease in tumor volume compared to the bacterial monotherapy group. By the sixth day, while the tumor volume of the bacterial monotherapy group increased steadily, the tumor volume of the combination therapy group continued to decrease and even achieved complete healing. Thus, our data demonstrated that combination therapy not only reduced the rate of tumor growth but also prevented the proliferation of tumor cells for an extended period.

Nowadays, various preclinical studies of BMTT have been conducted in different tumor models with promising results 69. The current major concern is how to guarantee the biosafety in short-term and long-term. In preclinical studies, blood routine indexes, blood biochemical values and H&E staining of organs were used to evaluate the short-term safety of BMTT 70, 71, so as our study. In fact, model strains in BMTT have been genetically modified for attenuating and constraining the growth in normal tissues. Moreover, second NIR irradiation in our study not only providing photothermal therapy for enhancing anti-tumor efficacy, but also achieving bacteria pyrolysis and protein denaturation for improving biosafety. Yet it is notable to avoid unexpected proliferation and spread of genetically modified bacteria. Early studies demonstrated that *E. coli* has shown unprecedented resistance to evolutionary escape, which laid a foundation for the long-term biosafety of BMTT 72. To date, several clinical phase Ⅰ studies were not detected significant systemic toxicity 73, 74. However, more consideration is required in human trials, including potential immune response, target delivery efficiency, and bacteria self-reproduction 75, 76. In conclusion, there still are numerous obstacles for BMTT on translation from preclinical studies to clinics usage.

Collectively, our newly developed multimodal imaged therapeutic bacteria strategy provides comprehensive diagnostic information about the tumor and enables monitoring capabilities during therapy, thereby facilitating the optimization of treatment plan. The introduction of therapeutic bacteria based on multimodal imaging combines several imaging modalities and advantages to ensure treatment safety. Combination therapy significantly reduces the likelihood of tumor recurrence while minimizing damage to normal tissues. This innovative approach offers a therapeutic paradigm for tumor treatment, characterized by high biosafety and therapeutic efficiency, which holds promise for future diagnostics and therapy.

## Supplementary Material

Supplementary figures.
