# Supplementary material for: Enhancing photothermal therapy of tumors with image-guided thermal control of gene-expressing bacteria
Source: Theranostics. 2024 Sep 9;14(15):5945–64. doi: 10.7150/thno.98257 (PMC11426242; doi:10.7150/thno.98257)
Supplement: Supplementary file 1 — Supplementary figures. [file thnov14p5945s1.pdf]

Enhancing photothermal therapy of tumors with image-guided thermal control of gene-expressing bacteria

Fengyi Zeng+[a,b], Meng Du+[a,b,c], Yaozhang Yang[a,b], Jinghui Fang[e], Yuanyuan Wang[d], MeeiChyn Goh[b], Yan Lin[e], Huaiyu Wang [f], Fei Yan*[d], and Zhiyi Chen*[a,b,c]

[a] Key Laboratory of Medical Imaging Precision Theranostics and Radiation Protection, College of Hunan Province, the Affiliated Changsha Central Hospital, University of South China

161 Shaoshan South Road, Changsha, Hunan Province (China)

[b] Institute of Medical Imaging, Hengyang Medical School, University of South China

28 Changsheng West Road, Hengyang, Hunan Province (China)

[c] Department of Medical Imaging, The Affiliated Changsha Central Hospital, Hengyang Medical School, University of South China

161 Shaoshan South Road, Changsha, Hunan Province (China)

[d] CAS Key Laboratory of Quantitative Engineering Biology, Shenzhen Institute of Synthetic Biology, Shenzhen Institute of Advanced Technology, Chinese Academy of Sciences

1068 Xueyuan Avenue, Shenzhen, Guangdong Province (China)

[e] Department of Ultrasound Medicine, Third Affiliated Hospital of Guangzhou Medical University

63 Duobao Road, Guangzhou, Guangdong Province (China)

[f] Center for Human Tissues and Organs Degeneration, Shenzhen Institutes of Advanced Technology, Chinese Academy of Sciences

1068 Xueyuan Avenue, Shenzhen, Guangdong Province (China)

[*] Corresponding Author E-mail: fei.yan@siat.ac.cn; zhiyi_chen@usc.edu.cn

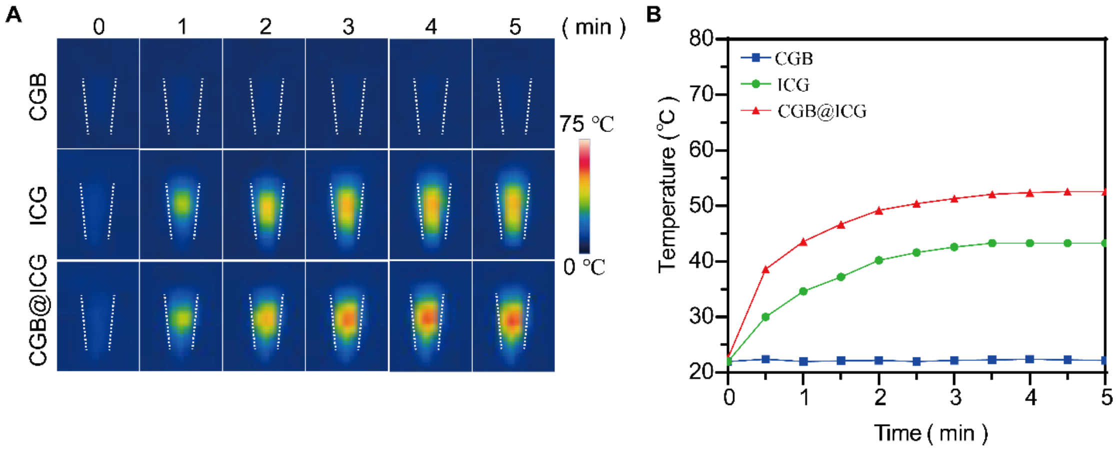

Figure S1. The *in vitro* photothermal conversion capability of components of CGB@ICG. (A) Infrared thermal images of CGB, ICG, and CGB@ICG exposed to 808 nm laser irradiation (1 W/cm^2) in 1.5 ml EP tube within 5 minutes. (B) The time-temperature curve of CGB, ICG, and CGB@ICG.

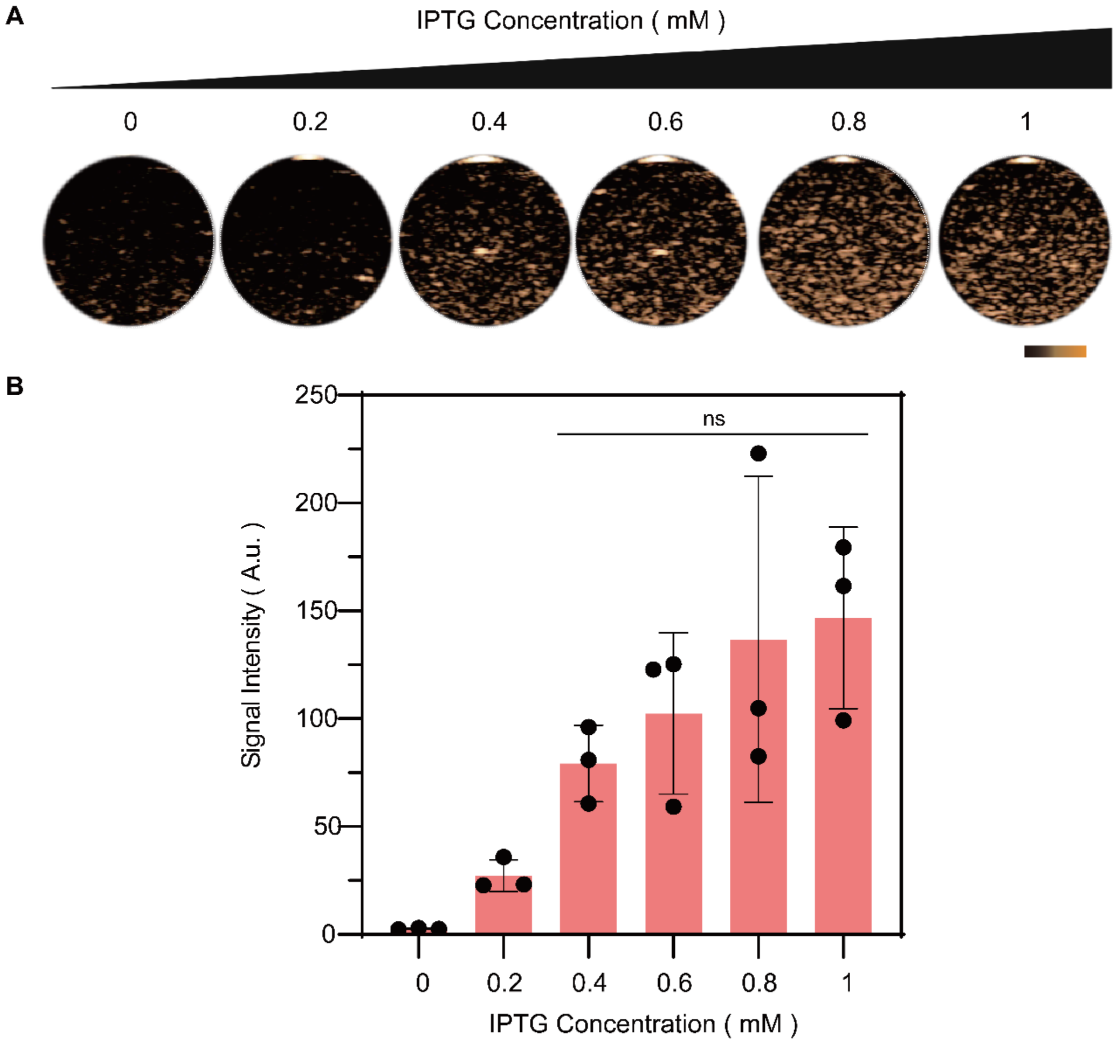

Figure S2. Dynamically regulated gene expression for *In vitro* ultrasound imaging of CGB@ICG. (A) Ultrasound images of CGB@ICG induced by a concentration series of IPTG (from 0.2 mM to 1 mM). (B) Quantitative analysis of ultrasound signal intensity of CGB@ICG.

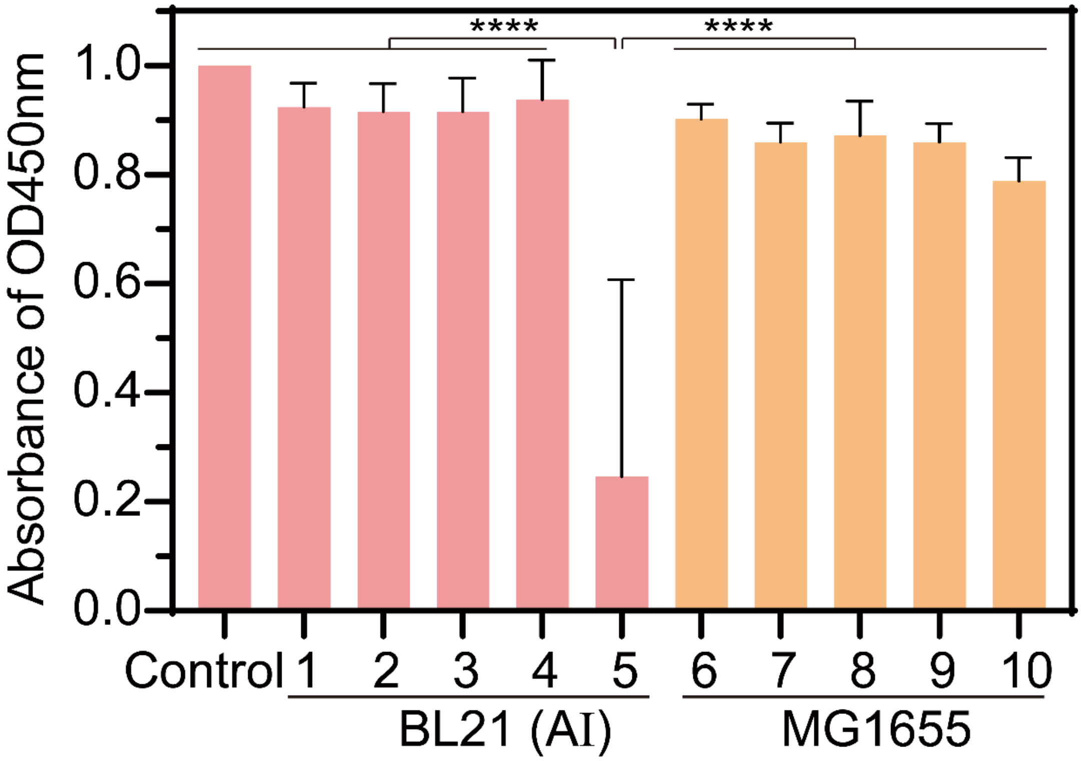

Figure S3. The in vitro cytotoxicity of ClyA protein expressed by different strains. Five colonies of each bacteria strain were selected and co-cultured with tumor cells. ClyA protein was then induced at the temperature of 42 °C, and the CCK-8 kit assay was used to evaluate the cytotoxicity.

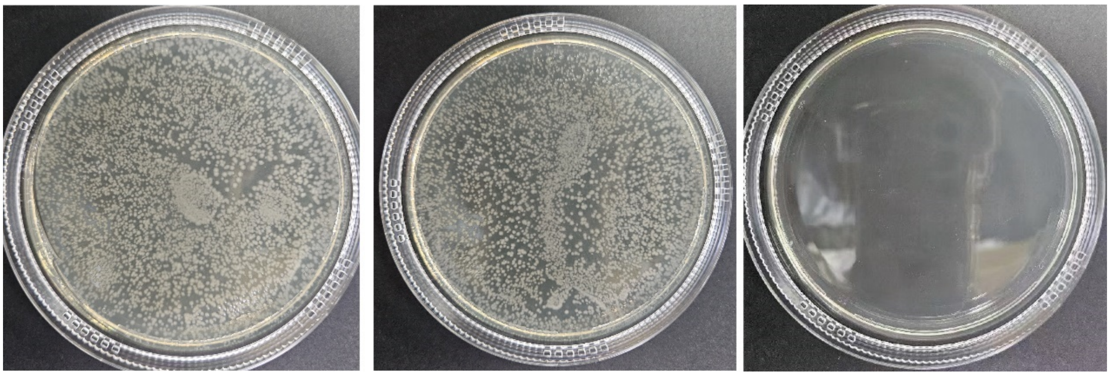

Figure S4. The cell viability of CGB@ICG in solid LB agar. The colonies of CGB@ICG treated at 30 ℃ for 20 minutes (left). The colonies of CGB@ICG treated at 42 ℃ for 20 minutes (middle) or at 50 ℃ for 10 minutes (right).

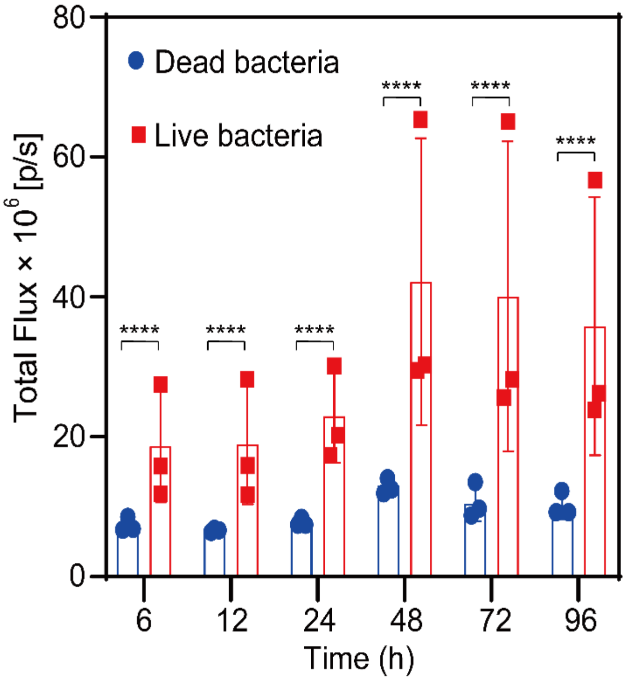

Figure S5. The total fluorescence intensity of tumor after intravenously injected dead and live bacteria at various time points. Data are presented as mean ± SD (n=3, $^{****}p< 0.0001$), and two-way ANOVA was performed using GraphPad Prism.

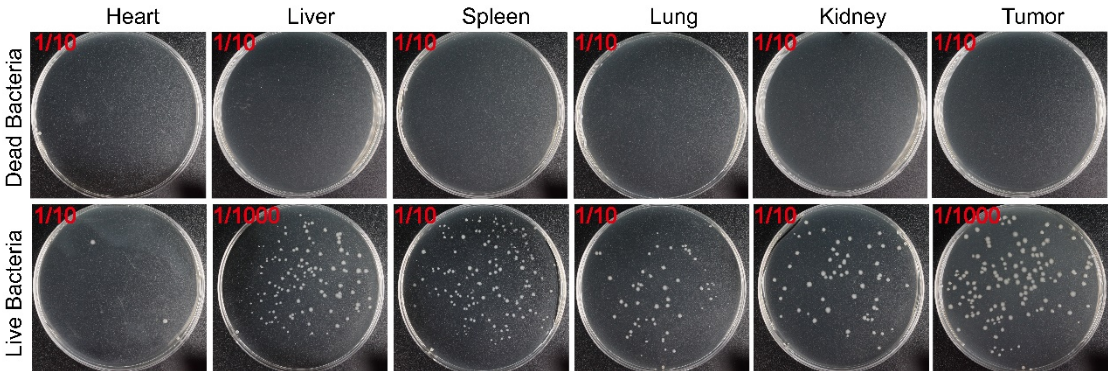

Figure S6. Photographs of solid LB agar plates of bacterial colonizes collected from isolated major organs and tumor tissues after 48 h injection dead and live bacteria group.

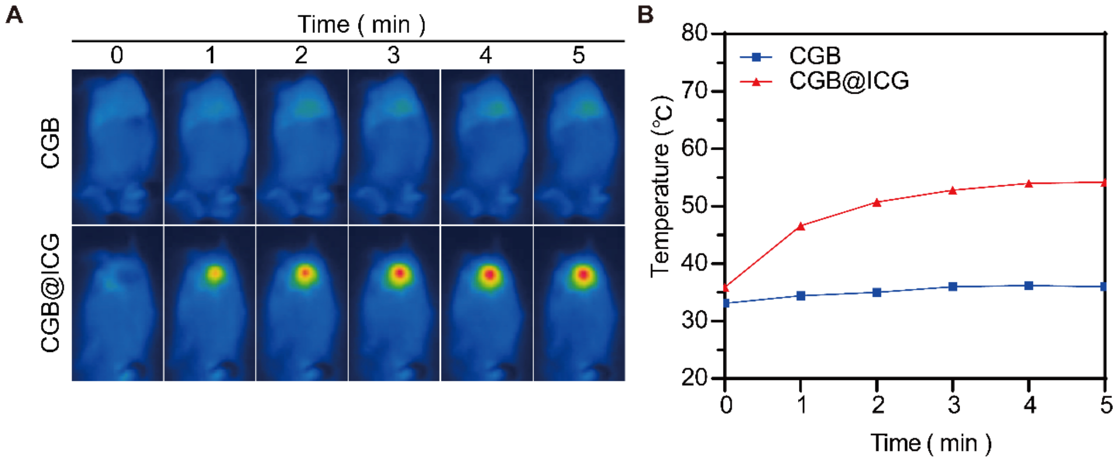

Figure S7. The photothermal conversion capability of CGB and CGB@ICG in tumor. (A) Infrared thermal of tumor exposed to 808 nm laser irradiation (1 W/cm^2) within 5 minutes after intravenous injection CGB or CGB@ICG. (B) The tumor time-temperature curve of CGB and CGB@ICG.

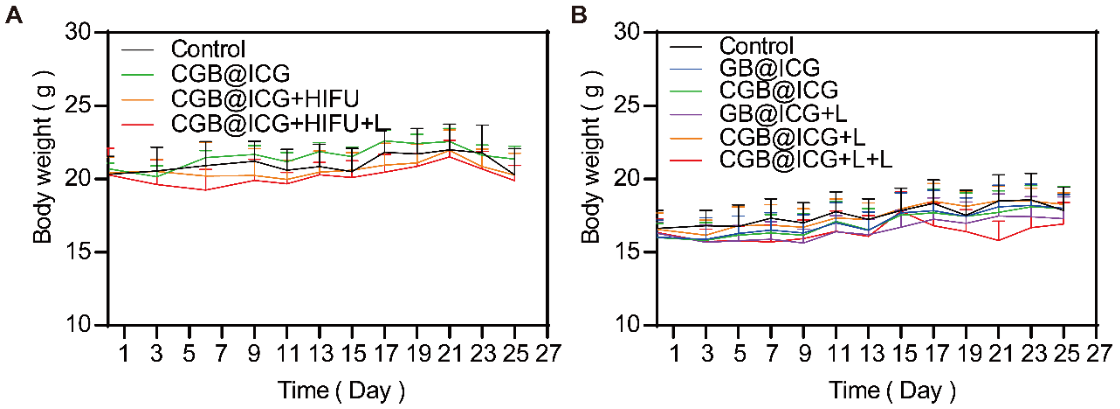

Figure S8. The body weight after various therapeutic treatments within 25 days. (A) The body weight change of control, CGB@ICG, CGB@ICG+HIFU, and CGB@ICG+HIFU+L. (B) The body weight change of control, GB@ICG, CGB@ICG, GB@ICG+L, CGB@ICG+L, and CGB@L+L.

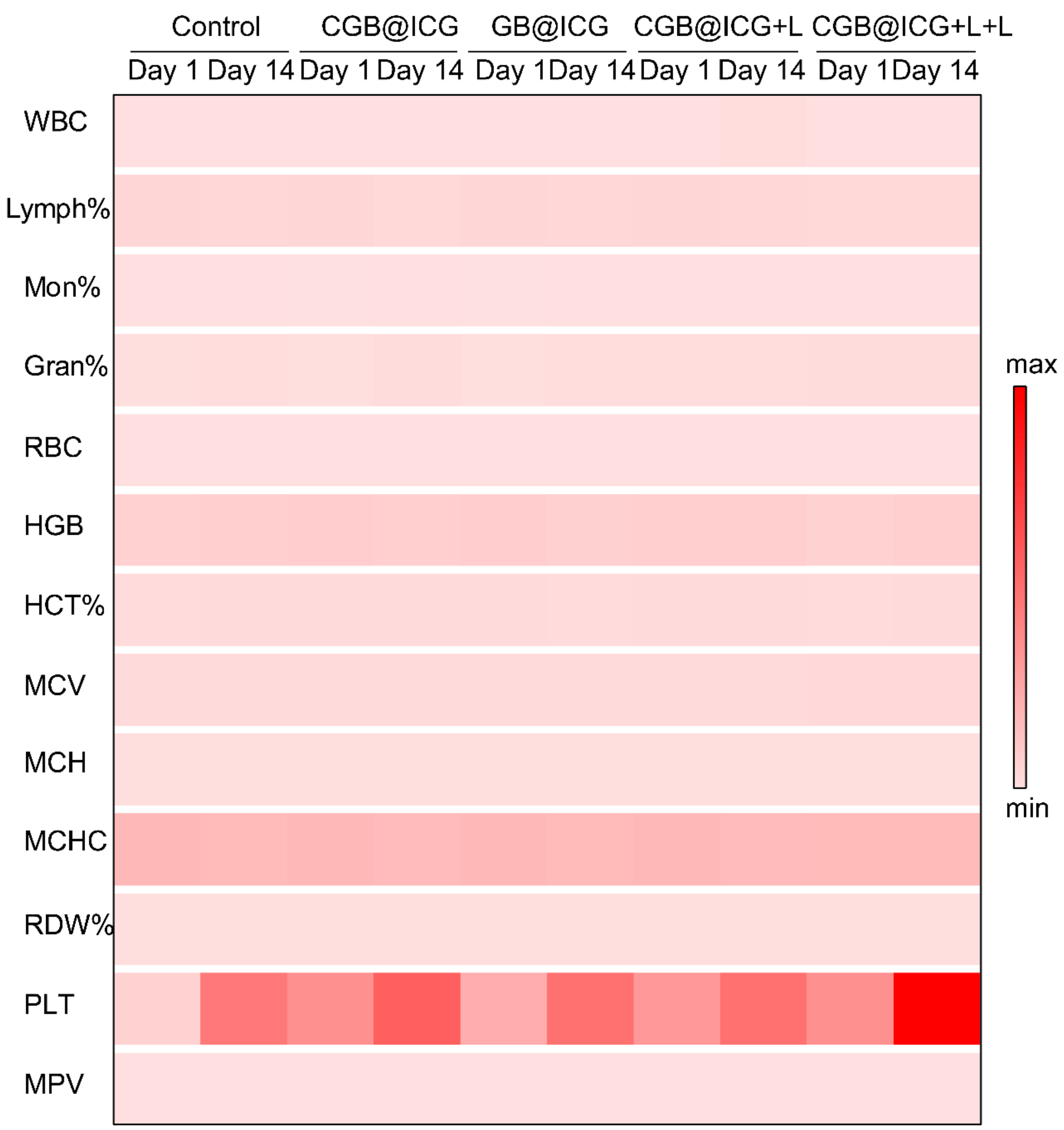

Figure S9. Routine blood tests of blood collected from mice at two timing points after intravenously injection of bacteria treated with different therapeutic treatments.

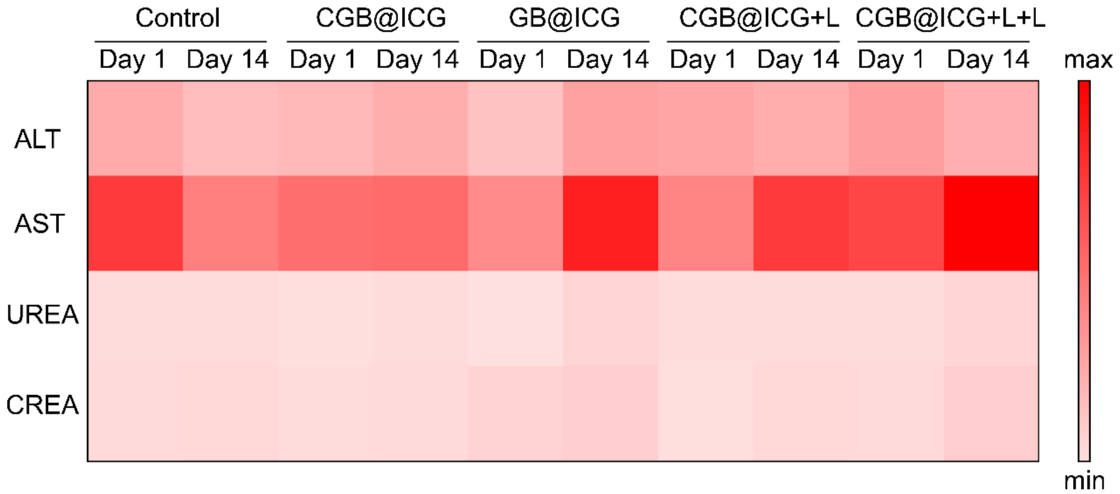

Figure S10. Blood biochemistry analysis of blood collected from mice at two timing points after intravenously injection of bacteria treated with different therapeutic treatments.

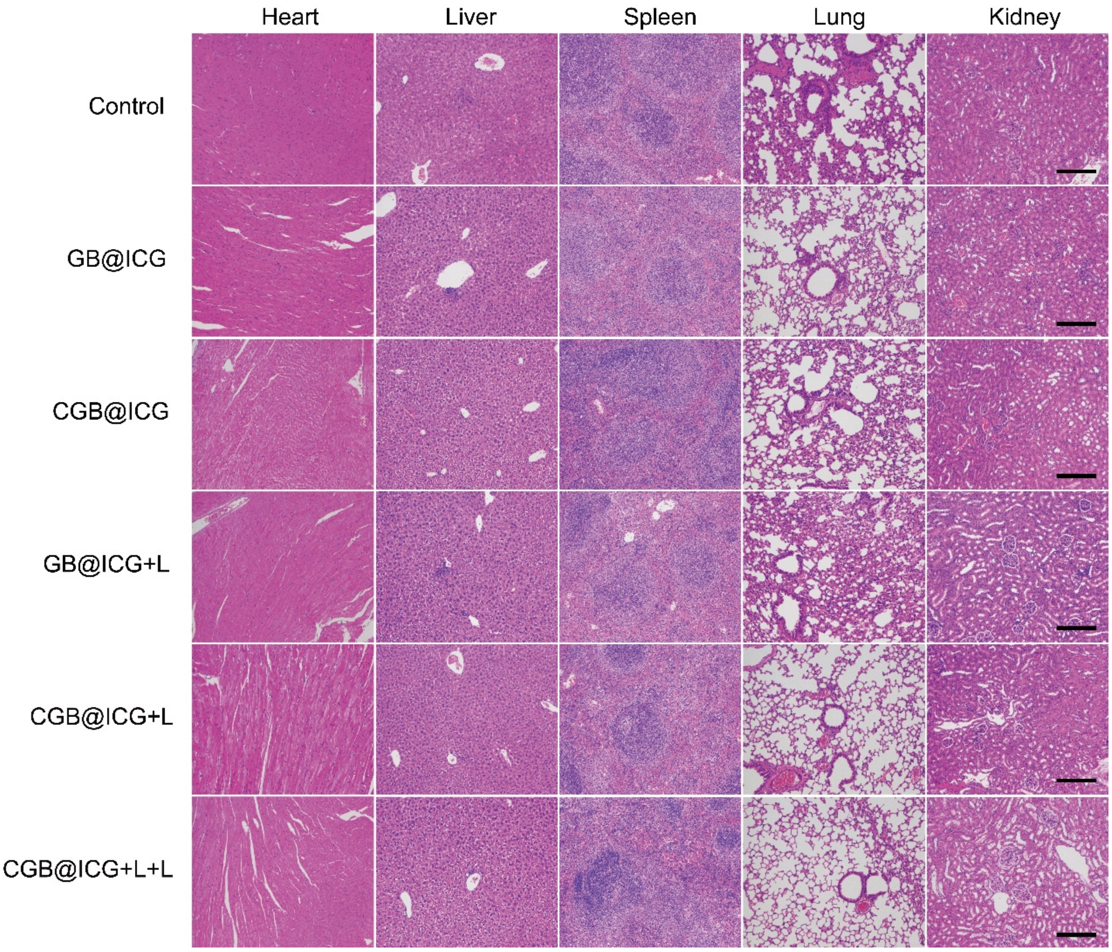

Figure S11. H&E staining of major organs collected from six groups of control, GB@ICG, CGB@ICG, GB@ICG+L, CGB@ICG+L, CGB@ICG+L+L (Scare bar, 200 μm).
